# The Stilbene Synthase Family in *Arachis*: A Genome-Wide Study and Functional Characterization in Response to Stress

**DOI:** 10.3390/genes14122181

**Published:** 2023-12-05

**Authors:** Ana Cristina Miranda Brasileiro, Marcos Aparecido Gimenes, Bruna Medeiros Pereira, Ana Paula Zotta Mota, Matheus Nascimento Aguiar, Andressa Cunha Quintana Martins, Mario Alfredo Saraiva Passos, Patricia Messenberg Guimaraes

**Affiliations:** 1 Embrapa Genetic Resources and Biotechnology, Brasília 70770-917, DF, Brazil; marcos.gimenes@embrapa.br (M.A.G.); bruagro6@gmail.com (B.M.P.); anazottamota@gmail.com (A.P.Z.M.); matheusnaguiar@gmail.com (M.N.A.); andressa.cqm@gmail.com (A.C.Q.M.); mario.saraiva@embrapa.br (M.A.S.P.); patricia.guimaraes@embrapa.br (P.M.G.); 2 National Institute of Science and Technology-INCT PlantStress Biotech-Embrapa, Brasília 70770-917, DF, Brazil

**Keywords:** abiotic stress, biotic stress, chalcone synthase, functional analysis, gene expression, peanut, resveratrol

## Abstract

Peanut (*Arachis hypogaea*) and its wild relatives are among the few species that naturally synthesize resveratrol, a well-known stilbenoid phytoalexin that plays a crucial role in plant defense against biotic and abiotic stresses. Resveratrol has received considerable attention due to its health benefits, such as preventing and treating various human diseases and disorders. Chalcone (CHS) and Stilbene (STS) Synthases are plant-specific type III Polyketide Synthases (PKSs) that share the same substrates and are key branch enzymes in the biosynthesis of flavonoids and stilbenoids, respectively. Although resveratrol accumulation in response to external stimulus has been described in peanut, there are no comprehensive studies of the CHS and STS gene families in the genus *Arachis*. In the present study, we identified and characterized 6 CHS and 46 STS genes in the tetraploid peanut and an average of 4 CHS and 22 STS genes in three diploid wild species (*Arachis duranensis, Arachis ipaënsis* and *Arachis stenosperma*). The CHS and STS gene and protein structures, chromosomal distributions, phylogenetic relationships, conserved amino acid domains, and *cis*-acting elements in the promoter regions were described for all *Arachis* species studied. Based on gene expression patterns of wild *A. stenosperma* STS genes in response to different biotic and abiotic stresses, we selected the candidate *AsSTS4* gene, which is strongly induced by ultraviolet (UV) light exposure, for further functional investigation. The *AsSTS4* overexpression in peanut hairy roots significantly reduced (47%) root-knot nematode infection, confirming that stilbene synthesis activation in transgenic plants can increase resistance to pathogens. These findings contribute to understanding the role of resveratrol in stress responses in *Arachis* species and provide the basis for genetic engineering for improved production of valuable secondary metabolites in plants.

## 1. Introduction

Chalcone (CHS) and Stilbene (STS) Synthases are enzymes belonging to the plant type III Polyketide Synthases (PKSs) superfamily that share the same substrates (one molecule of *p*-coumaroyl-CoA and three molecules of malonyl-CoA) to catalyze the first committed step of the flavonoid and stilbenoid biosynthesis pathways, respectively [1]. Resveratrol (trans-3,5,4′-trihydroxystilbene) is the best-known and the most studied stilbene compound due to its broad range of beneficial biological activities, such as antioxidant, antimicrobial, antiviral, anticancer, antidiabetic, anti-inflammatory, and cardio- and neuroprotective activities [2]. These health-promoting functional properties make resveratrol a promising natural agent for preventing and treating general health and various diseases and disorders. In addition, as a phytoalexin, resveratrol is elicited and accumulated in response to several biotic and abiotic stresses to protect plants against pathogen infection, herbivore attack, ultraviolet (UV) light irradiation, mechanical damage, heavy metals, treatments with chemicals, and others [3]. Due to its current relevance in the pharmaceutical, nutraceutical, food, veterinary, and cosmetic industries, large-scale resveratrol production is urgently required to meet the world’s market demand and new technologies should be developed to improve its extraction from plants [4,5].

CHS and STS share extensive similarities in their amino acid sequences, and evolutionary studies suggest that STS genes have evolved from CHS genes more than once in a lineage-specific manner through neofunctionalization [6,7,8]. However, unlike CHS, which is ubiquitously present in all higher plants, STS is restricted to a limited number of stilbene-producing plants, comprising 34 phylogenetically distant botanical families that include gymnosperms and angiosperms (monocots and dicots) [9].

*Arachis* species (Fabaceae) are among these few plants that naturally synthesize resveratrol, and despite the advances in exploring this molecule, only the cultivated species (*Arachis hypogaea* L.) has been considered for producing this compound [10]. In the last few years, however, the knowledge about resveratrol synthesis and metabolism in wild *Arachis* species belonging to distinct botanical sections has been expanded [11,12,13,14,15,16]. Our studies demonstrated that some wild species produced higher levels of resveratrol, associated with the induced expression of STS genes, than cultivated species upon UV exposure. In particular, *A. lignosa* L. (section *Procumbentes*), *A. triseminata* L. (section *Triseminatae*), *A. duranensis* L., *A. stenosperma* L., and *A. ipaënsis* L. (section *Arachis*) were identified as potential novel rich sources of resveratrol due to their high levels content, adding value to these overlooked genetic resources [11,12]. These findings open new opportunities to explore wild *Arachis* species directly as natural sources for resveratrol bioproduction or as donors of *STS* genes for resveratrol metabolic engineering in heterologous microorganisms or non-stilbene-producing plants [4,17].

Until recently, grapevine (*Vitis* spp.) and mulberry (*Morus notabilis* C.K. Schneid) were the only stilbene-producing plants for which a comprehensive characterization was conducted for the STS multigenic family consisting of closely related paralogs [18,19,20]. In this respect, the availability of the complete genome sequences of cultivated peanut (*A. hypogaea*) and their wild progenitors (*A. duranensis* and *A. ipaënsis*) [21,22,23,24,25] represents an excellent opportunity to advance in the knowledge of the organization, function, and evolution of the STS gene family in these stilbene-producing species. Furthermore, the recent release of the first draft genome sequence for the highly pathogen-resistant *A. stenosperma* (http://peanutbase.org) has greatly facilitated genome-wide studies in the genus.

In the present study, a genome-wide analysis was undertaken to characterize CHS and STS multigenic families in the genomes of four *Arachis* species. We identified 6 CHS and 46 STS genes in the tetraploid cultivated *A. hypogaea* and an average of 4 CHS and 22 STS genes in the diploid wild *A. duranensis*, *A. ipaënsis*, and *A. stenosperma*. Gene and protein structures, chromosomal distribution patterns, phylogenetic relationships, conserved amino acid domains, and *cis*-acting elements in the promoters were characterized in each species. Additionally, transcriptome analysis showed that *A. stenosperma* STS genes exhibited dynamic expression patterns in response to different biotic and abiotic stresses and revealed *AsSTS4* as a promising candidate gene for genetic engineering towards enhanced resistance to pathogens. For *in planta* functional validation, *AsSTS4* was cloned and successfully overexpressed in hairy roots of a peanut cultivar susceptible to the root-knot nematode (RKN) *Meloidogyne arenaria*, leading to a significant reduction of 47% in the number of nematode galls. To our knowledge, this is the first study describing the overexpression of a plant STS gene to enhance nematode resistance, pointing out *AsSTS4* as a valuable candidate gene to be exploited in future genetic breeding programs.

## 2. Results

### 2.1. Identification and Characterization of CHS and STS Genes in Arachis spp.

Genome-wide searches were performed based on the presence of conserved CHS and STS domains on the genome of four *Arachis* species: *A. duranensis* (2n), *A. ipaënsis* (2n), *A. stenosperma* (2n), and *A. hypogaea* (4n). An average of 37 genes were identified as CHS- and STS-coding putative proteins in diploid species and 74 in the tetraploid, with 27–32% being pseudogenes, i.e., sequences containing partial CHS/STS domains, a premature stop codon, or frameshift mutations (Table 1). After manually removing redundancies, only proteins containing both the chalcone/stilbene synthases N- (PF00195) and C-terminal (PF02797) domains were kept. Proteins were then annotated as CHS or STS according to the presence of conserved residues around Met98 and Thr132 [26] as well as the nine residues recently predicted to be under positive selection in peanut by [6]. Each gene family was named based on the chromosome locations for each species.

**Figure 1:**
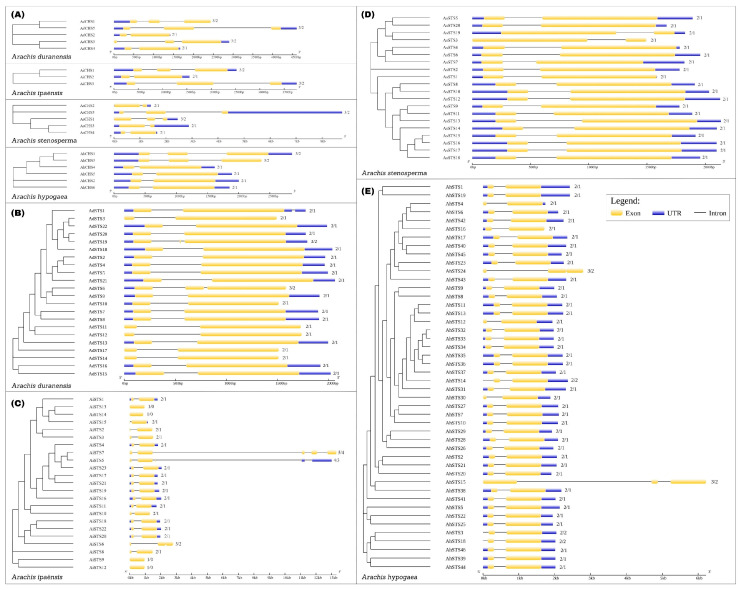
Exon–intron gene structure of CHS (**A**) and STS in *Arachis duranensis* (**B**); *A. ipaënsis* (**C**); *A. stenosperma* (**D**); and *A. hypogaea* (**E**).

As a result, we identified 5 CHS and 22 STS in *A. duranensis*, 3 CHS and 23 STS in *A. ipaënsis*, 5 CHS and 20 STS in *A. stenosperma* and 6 CHS and 46 STS genes in *A. hypogaea* (Table 1). Similar to other plant PKS family genes [27,28,29], the 19 *Arachis* CHS exhibited either a 2-exon/1-intron (53%) or a 3-exon/2-intron (47%) architecture (Figure 1A). The great majority (90%) of the 111 STS genes was characterized by a very similar gene structure harboring a single intron and two exons in all *Arachis* species (Figure 1B–E). The intron and exon sizes of the STS family members are quite conserved within each species, with a few lacking annotated 5′UTR. Only five STS genes (*AdSTS6* and *19*; *AiSTS6*; *AhSTS15* and *24*) are the exception from this usual structure and exhibit a 3-exon/2-intron organization (Figure 1B,C,E). Some *A. ipaënsis* and *A. hypogaea* genes showed intron-exon structures that are uncharacteristic for STS genes (Figure 1C,E), which could be due to gene prediction problems, such as intronless (*AiSTS9*, *12*, *13* and *14*), multiple exons and introns (*AiSTS5* and *7*) or start codon introns (*AhSTS3*, *14* and *18*). Detailed characteristics of CHS and STS genes in the four *Arachis* species are shown in Tables S1–S4.

The predicted proteins encoded by *Arachis* CHS and STS genes ranged from 293 to 433 amino acids with an average of 383. The molecular weight (MW) varied from 31.80 to 48.14 kDa, while the theoretical isoelectric point (p*I*) values were between 5.17 and 7.18. Overall, other physicochemical properties, such as instability index, aliphatic index, and GRAVY, are similar among the *Arachis* CHS and STS proteins. The subcellular localization revealed that plastid (53%) and cytoplasm (42%) are the most frequent sites predicted for the 19 *Arachis* CHS proteins. In contrast, the great majority (82%) of the 111 *Arachis* STS proteins are predominantly localized in the plastid, with only a few found to be cytoplasm-localized (17%), indicating that the subcellular localization of the STS family proteins is highly conserved in the *Arachis* species studied. Interestingly, the plastid is considered a major compartment to produce defense-related signaling molecules. Apart from cytoplasm and plastid, members of CHS and STS were also found in relatively low distribution (5 and 1%, respectively) in the Golgi apparatus. Detailed information about the physicochemical properties and the subcellular localization of CHS and STS proteins in the four *Arachis* species are shown in Tables S1–S4.

### 2.2. Chromosomal Distribution and Organization of Arachis CHS and STS Genes

**Figure 2:**
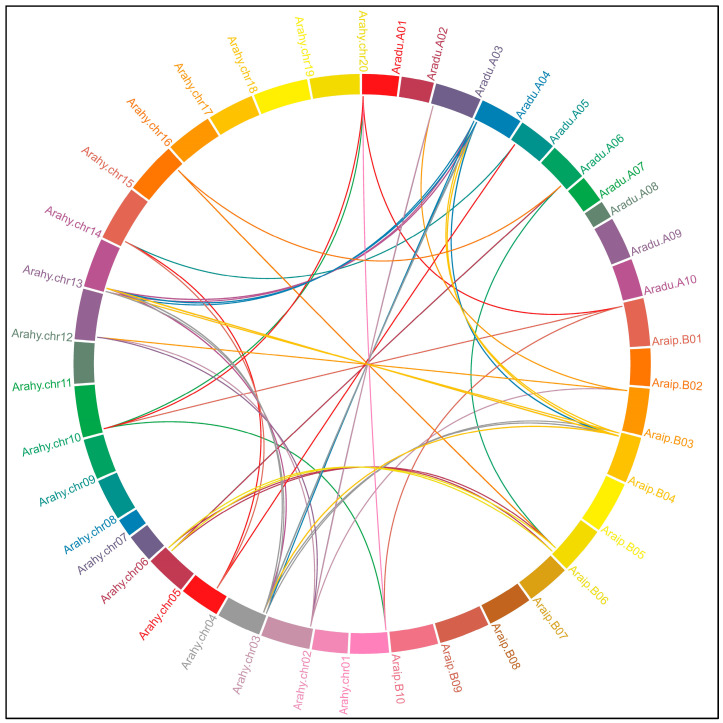
Genomic distribution and syntenic relationships of the CHS and STS genes in *Arachis* spp. The ten chromosomes of *A. duranensis* is represented as Aradu (A01 to A10) and *A. ipaënsis* as Araip (B01 to B10), and the 20 chromosomes of *A. hypogaea* as Arahy (chr01 to chr20). The syntenic relationships between the genes are represented by lines.

The genome localization and syntenic relationships of the CHS and STS genes were predicted for *A. duranensis*, *A. ipaënsis*, and *A. hypogaea. A. stenosperma* was not included in this analysis since the single whole genomic sequence available for this species does not yet have a gene structure annotation. The chromosomal location of the CHS genes showed that they were unevenly distributed on chromosomes 03, 04, 05, and 06 of the wild species and chromosomes 03, 05, 13, 14, and 15 of the cultivated species (Figure 2). Conversely, the great majority of the *Arachis* STS genes were grouped in gene clusters on the chromosome 04 of each diploid wild species (86%) and the collinear chromosomes 04 and 14 (85%) of the cultivated tetraploid species (Figure 2; Table 1). The few remaining STS genes were unevenly distributed on chromosomes 01 and 06 of the wild species and on chromosomes 01, 11, and 16 of the cultivated species. Moreover, most of the *Arachis* CHS and STS genes were located on the distal chromosomal regions, typical for the gene-rich characteristic of these recombination hotspots, as previously observed for other *Arachis* gene families [30,31,32]. No CHS and STS representatives were found for any species on chromosomes 02, 07 to 10, 12, and 17 to 20.

**Figure 3:**
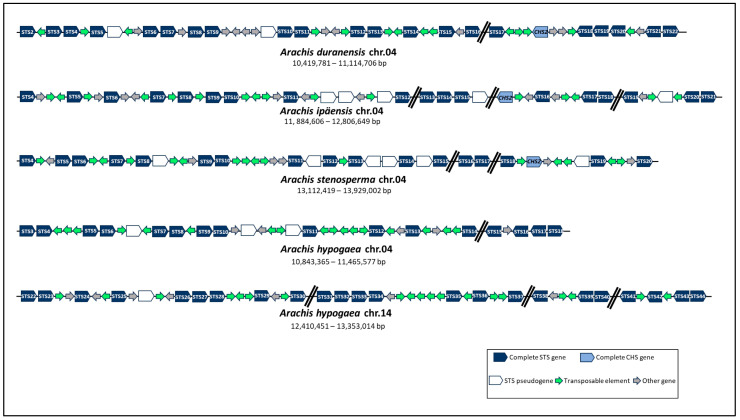
Schematic representation of STS gene clusters organization on chromosomes 04 and 14 of *Arachis* spp. Right and left arrows indicate whether genes or transposable elements are located on the + or − strand, respectively.

For a more comprehensive investigation of the STS gene clusters in *Arachis*, we further analyzed their physical organization in chromosome 04 of diploid species and collinear chromosomes 04 and 14 of tetraploid one. The distribution and orientation of functional genes (STS, CHS, and others), pseudogenes, and transposable elements (TEs) were arranged in each cluster (Figure 3). In all three diploid wild species, STS genes were positioned in a single aligned gene cluster on each chromosome 04, harboring 17 to 21 complete STS genes occurring within less than 922 kb apart, with an average density of 2.4 genes per 100 kb (Table 1; Figure 3). Likewise, in the cultivated tetraploid species, 16 and 23 complete STS genes were identified on chromosomes 04 (622 kb) and 14 (943 kb), respectively, with an average density of 2.5 genes per 100 kb (Table 1; Figure 3). In all species studied, these complete STS genes are irregularly spaced within each cluster and often interrupted by STS pseudogenes and other functional genes encoding non-STS proteins (Figure 3). Moreover, *Arachis* STS gene clusters are also characterized by the presence of various TEs, with an average of 3.3 TEs per 100 kb (Figure 3), predominantly from retrotransposons classes LTR (42.6%) and non-LTR/LINE (15.9%) and DNA transposons superfamilies CACTA (15.3%) and MULE (12.4%), in accordance to [21].

### 2.3. Phylogenetic Analysis of CHS and STS Proteins

**Figure 4:**
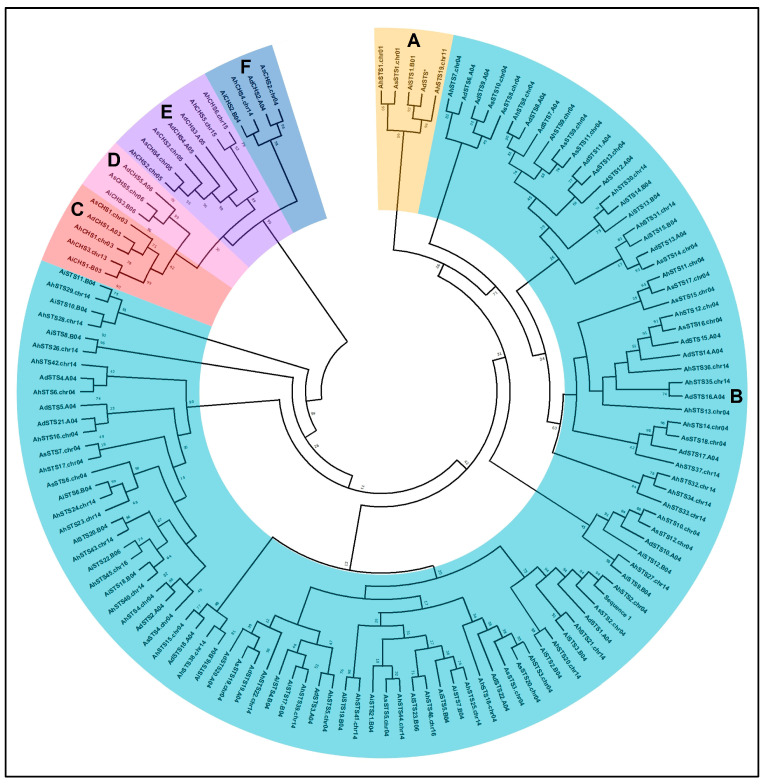
Phylogenetic tree of STS (Groups **A** and **B**) and CHS (Groups **C**, **D**, **E**, and **F**) S amino acid sequences from *Arachis duranensis*; *A. ipaënsis*; *A. stenosperma*; and *A. hypogaea*.

To explore the evolutionary relationships among the *Arachi*s CHS and STS proteins, a phylogenetic tree was built based on the amino acid sequences encoded by 27 *A. duranensis* (5 CHS, 22 STS), 26 *A. ipaënsis* (3 CHS, 23 STS), 25 *A. stenosperma* (5 CHS, 20 STS), and 52 *A. hypogaea* (6 CHS, 46 STS) genes (Table 1). The phylogenic analysis showed that proteins belonging to the *Arachis* CHS and STS families formed two clearly separate major clades with a high bootstrap support (Figure 4).

The CHS sequences were distributed into four subclades, each formed by proteins encoded by genes with similar positions on chromosomes 03/13, 04/14, 05/15, and 06 of each diploid and tetraploid species (Figure 4). The STS sequences retrieved from the four *Arachis* species were grouped in several subclades, with sequences of each species spread into different clades having higher similarity to other species sequences than to those of the same species, as previously observed by our group [11]. While all STS proteins encoded by genes on chromosomes 01 and 11 were grouped together, those encoded by the 103 genes located on chromosomes 04 and 14 were more diverse and included the four STS genes from *A. ipaënsis* and *A. hypogaea* located, respectively, on chromosomes 06 and 16.

Overall, CHS and STS subclades comprise proteins from the four *Arachis* species located in similar positions on the chromosomes. For instance, some subclades are formed by proteins encoded by genes with sequential positions on chromosome 04 of the different species, like that comprising *AhSTS7*, *AdSTS6*, *AdSTS9*, *AsSTS8*, and *AsSTS10*. Likewise, subclades are formed by proteins whose genes have similar positions on chromosomes 04 and 14 but are located outside of the STS gene clusters (Figure 3), like the one comprised of *AhSTS2*, *AsSTS2*, *AdSTS1*, *AiSTS2*, and *AiSTS3*, (chromosome 04) and *AhSTS20* and *AhSTS21* (chromosome 14; Figure 4). Interestingly, the subclade formed entirely by STS proteins located at chromosomes 01 (*AhSTS1*, *AsSTS1*, and *AiSTS1*) and 11 (*AhSTS19*), also includes an STS pseudogene from *A. duranensis* (AdSTS*; Figure 4). Conversely, a unique subclade comprises sequences (*AiSTS10*, *AiSTS11*, *AhSTS28*, and *AhSTS29*) coding by genes exclusively located on the STS clusters in chromosomes 04 and 14. Vannozzi et al. [18] also observed that grapevine STS proteins encoded by genes located on different chromosomes formed separate clades. Here, only few clades are composed by *Arachis* proteins encoded by genes located on different chromosomes, such as *AhSTS4* located at the STS cluster in chromosome 04 that was grouped with *AhSTS40* and *AhSTS43* located on the chromosome 14, and AhSTS45 on chromosome 16.

To better understand the evolution of CHS and STS families in legumes, we also performed a phylogenetic analysis based on the gene sequences of three *Arachis* species (*A. duranensis*, *A. ipaënsis*, and *A. hypogaea*) and eleven legume species for which the complete annotated genome is available at LIS (Legume Information System) database, using four non-legume species as outgroups (Figure S1). As observed with the phylogenetic tree based on *Arachis* amino acid sequences (Figure 4), the legume gene tree shows the generally expected phylogenetic relationships, with CHS and STS homologs falling in two distinct clades. The first large clade is composed entirely of STS syntenic genes from the three *Arachis* species, indicating that, among the major crop and model legume species examined here, *Arachis* is the only harboring STS genes. The second large clade is formed exclusively by CHS syntenic genes from all legume and non-legume species, including the three *Arachis*, mixed in distinct subclades, confirming that the CHS gene family is ubiquitous in higher plants. These results support that *Arachis* STS genes have independently evolved different mutations from the typical CHS genes in the ancestor of Fabaceae, as recently reported by [6].

### 2.4. Conserved Functional Domain and Motifs Analysis

The type III PKS active site residues of the enzymes and CHS/STS signature motif (‘WGVLFGFGPGLT’; [33]) were conserved among the *Arachis* CHS and STS proteins, which share an average of 90.7% sequence identity without significant insertions or deletions (Figure S2). In accordance with [26], the *Arachis* STS proteins contain 11 unvaried amino acid residues surrounding Met98 (‘EDMMIREVPRV’) and Thr132 (‘CTTSGVALPGV’) (Figure S2), as described for the peanut STS1 protein (ID AB027606). Likewise, at the same positions, *Arachis* CHS proteins show the consensus sequences ‘QDMVVVEVPRL’ and ‘CTTSGVDMPGA’ (Figure S2) that are highly conserved residues among the members of the CHS superfamily in distinct plant species such as alfalfa, pine, and sweet orange [26,34]. The nine positively selected sites of typical peanut STS, as recently predicted by [6], were also present in all *Arachis* species. Previous studies indicate that differences in a few amino acid residues in these conserved residues can lead to variations in the crystal structures and enzymatic activities or modifications in the loop regions among the type III PKS [1,6] and allow for the distinction of CHS and STS family members in the four *Arachis* species.

**Figure 5:**
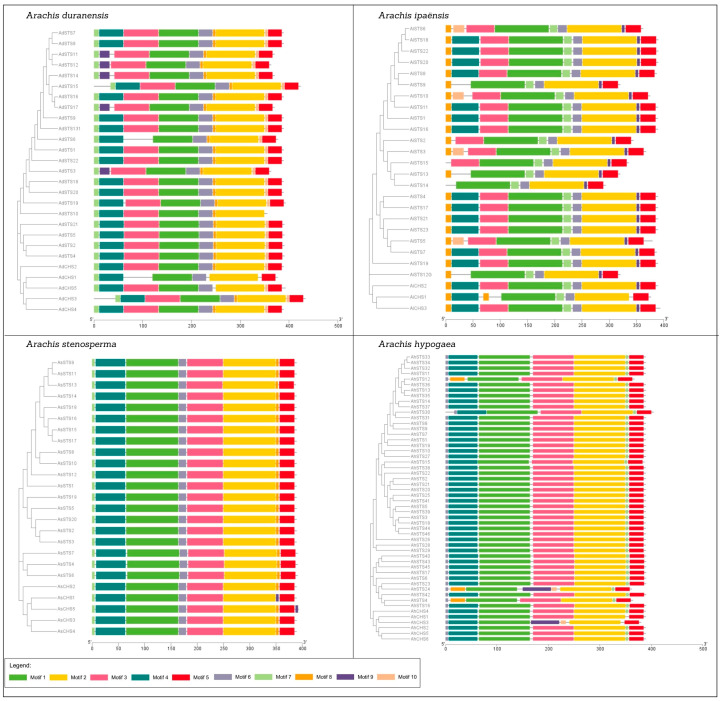
Protein structure of CHS and STS in *Arachis duranensis*; *A. ipaënsis*; *A. stenosperma*; and *A. hypogaea*.

**Figure 6:**
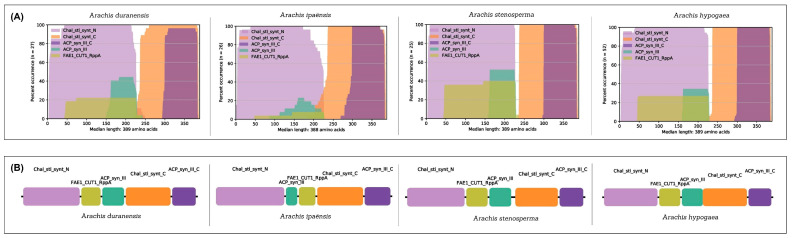
Protein organization of CHS and STS in *Arachis duranensis*; *A. ipaënsis*; *A. stenosperma*; and *A. hypogaea*. (**A**) Percentage of occurrence and (**B**) organization of conserved protein domains in predicted proteins.

The distribution, position, and frequency of occurrence of PFAM domains showed a highly conserved protein structure across the CHS and STS families in *Arachis* spp. (Figure 5 and Figure 6), consisting of the chalcone and stilbene synthases N-terminal (PF00195; Motifs 1, 3, and 4) and C-terminal (PF02797; Motifs 2 and 5) domains, both used here to identify CHS and STS proteins. In addition, these proteins also share three common domains, FAE1_CUT1_RppA (PF08392), ACP_synthase_III (PF08545), and ACP_synthase_III_C (PF08541) (Figure 6), which are characteristic features of type III PKSs in plants and are involved with lipid metabolism [35].

### 2.5. Cis-Acting Elements in CHS and STS Genes Promoter Regions

The 1500 bp sequences upstream of the first nucleotide of the 5’UTR could be extracted from all *A. hypogaea* (52) and *A. stenosperma* (25) CHS and STS genes. However, only 20 sequences were retrieved from the 27 *A. duranensis* genes and 11 out of 26 from *A. ipaënsis* due to the significant number of unspecified nucleotides in their respective promoter region sequences. A total of 93 *cis*-acting elements could be predicted in these 108 putative promoter sequences of CHS and STS genes from all four *Arachis* species, where the most common were the well-characterized TATA-box (49%) and CAAT-box (21%), which are considered core elements essential for eukaryotic promoter activity. Two other TATA elements (AT~TATA-box and TATAAAAT) were also frequently found (6.9 and 1.3%, respectively) in many promoters. The remaining *cis*-acting elements were clustered into four categories according to their putative roles and involvement in distinct pathways: 17 responsive to hormones (HRE), 28 to light (LRE), 18 to stresses (STRE), and 14 related to tissue specificity and development (TS&DEV). Although some promoters are responsive to more than one stimulus, here, they were classified based on only one response. The other 12 sequences identified as putative promoter elements (A-box, CCGTCC-motif, JERE, CTAG-motif, box S, OCT, DRE core, E2Fb, GC-motif, HD-Zip 3, AACA_motif and CARE) had no information about their functions in PlantCARE database and therefore were not further analyzed.

The total and mean number of putative *cis*-elements classified in the four categories (HRE, LRE, STRE, and TS&DEV) varied among the four *Arachis* species and the gene family (Table 2). In the tetraploid *A. hypogaea*, the total number of *cis*-elements found in the STS promoters was at least two times higher than the observed in the wild diploid species. Likewise, a larger number (>1.4 times) of elements was also observed in the CHS promoters of the tetraploid species compared to the diploids *A. duranensis* and *A. stenosperma*, whereas *A. ipaënsis* has only one CHS promoter identified due to errors in DNA sequences. When the mean number of *cis*-elements per promoter was taken into account for each species instead of the total number, the ratio tetraploid:diploid species was close to one for both CHS and STS genes (Table 2).

**Figure 7:**
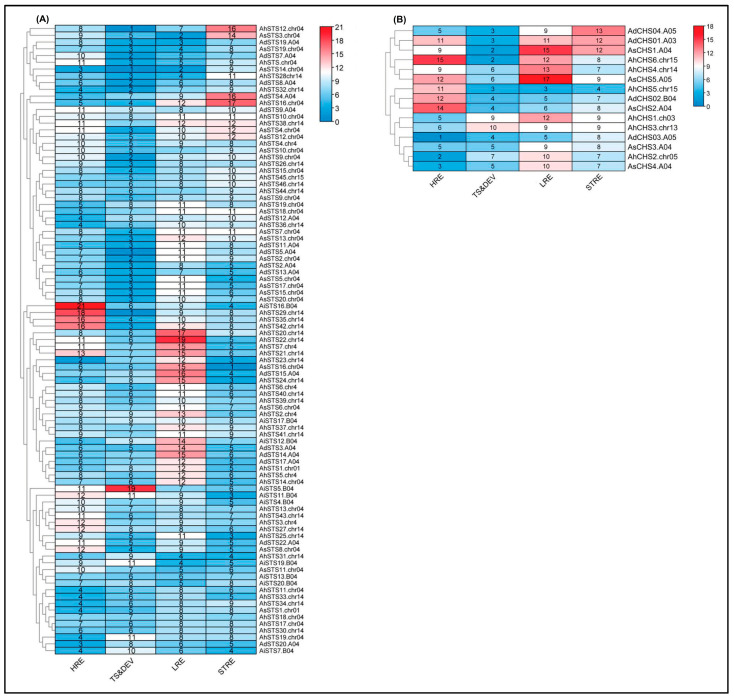
Heatmap of *cis*-acting elements associated with responses to hormones (HRE), light (LRE), and stress (STRE) and related to tissue specificity and development (TS&DE) in the promoter regions of STS (**A**) and CHS (**B**) genes of *A. duranensis* (Ad), *A. ipaënsis* (Ai), *A. stenosperma* (As), and *A. hypogaea* (Ah). The heatmap colors range from red to blue scale, where darker colors indicate increasing and decreasing values in the numbers of *cis*-elements.

Figure 7A,B shows the relationships between the STS and CHS genes, respectively, based on the observed numbers of *cis*-elements classified in the HRE, LRE, STRE, and TS&DEV categories. Regulatory elements belonging to the four categories were found in all putative promoter sequences analyzed, allowing the identification of promoter clusters based on the differences in the number of elements in each category. For instance, the first group (*AiSTS16*, *AhSTS29*, *AhSTS35*, and *AhSTS42*) showed the greatest number (>16) of elements related to hormone response (HRE), while a second group with eight sequences (*AhSTS20*, *AhSTS22*, *AhSTS7*, *AhSTS21*, *AhSTS23*, *AsSTS16*, *AdSTS15*, and *AhSTS24*) showed the highest number (>12) of light-responsive elements (LRE). Four genes (*AhSTS12* and *AsSTS3*, *AdSTS4*, and *AhSTS16*) formed two groups with the largest number (>14) of elements responsive to stress (STRE). No clear STS promoter clustering was observed concerning the numbers of *cis*-elements classified as related to tissue specificity and development (TS&DEV). We also found significant clustering based on the number of *cis*-elements in the 15 *Arachis* CHS gene promoters for HRE, LRE, and STRE categories (Figure 7B). Overall, CHS and STS promoter sequences were not clustered together based on the species. For instance, *A. hypogaea* promoters were distributed in all clusters, including the sequences located at chromosomes 04 and 14 (Figure 7).

**Figure 8:**
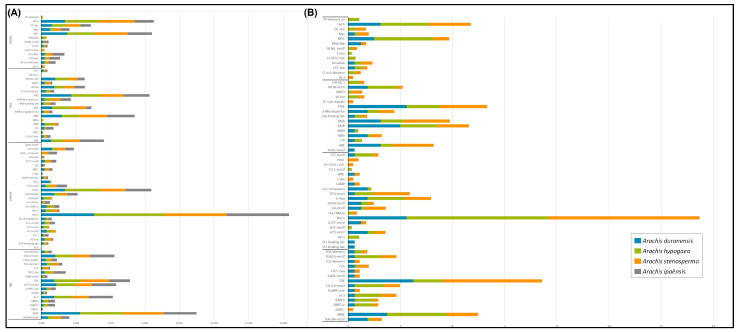
Distribution in the HRE, LRE, STRE, and TS&DEV categories of *cis*-acting elements found in the putative promoter sequences of STS (**A**) and CHS (**B**) genes from *A. duranensis*, *A. ipaënsis*, *A. stenosperma* and *A. hypogaea*.

Among the 17 hormone responsive (HRE) *cis*-elements found in STS promoter genes, the most frequent in all species were ABRE and ERE, involved in ABA and ethylene responses, respectively, and CGTCA- and TGACG-motif, both involved in methyl jasmonate (MeJA) responsiveness (Figure 8A). Light-responsive (LRE) is the category that shows the greatest number of *cis*-elements (28) in STS promoter regions, with Box 4 being highly represented followed by G-Box. In the stress category, STRE, MYB, ARE, WUN-motif and W-box were the most abundant *cis*-elements and similarly distributed in the STS gene promoters. In the tissue specificity and development category, promoter regions of STS genes were enriched with TATA, MYC, and O2-site elements that are also related to general stress responses. For the four functional categories (Figure 8B), CHS promoter regions were less diverse than those found in the STS genes with a total of 71 *cis*-elements found in the 15 promoter regions analyzed.

### 2.6. Arachis Stenosperma CHS and STS Gene Expression Patterns under Biotic and Abiotic Stresses

The expression patterns of the 20 genes encoding for STS and five for CHS in *A. stenosperma* were analyzed using our previous transcriptome surveys to investigate their transcriptional dynamics in response to biotic and abiotic stresses. The wild *A. stenosperma* was chosen for this expression analysis as it is a high-resveratrol producer, is adapted to marginal habitats, harbors stress-resilience traits, and is highly responsive to multiple environmental stimuli [36]. Transcriptome data were obtained from *A. stenosperma* plants submitted to different types of stresses: UV exposure; drought treatments in moderate (dry-down) and severe (dehydration) conditions; nematode infection (at 3, 6, 7, and 9 days after infection; DAI); and combined drought imposition and nematode infection (cross-stress).

**Figure 9:**
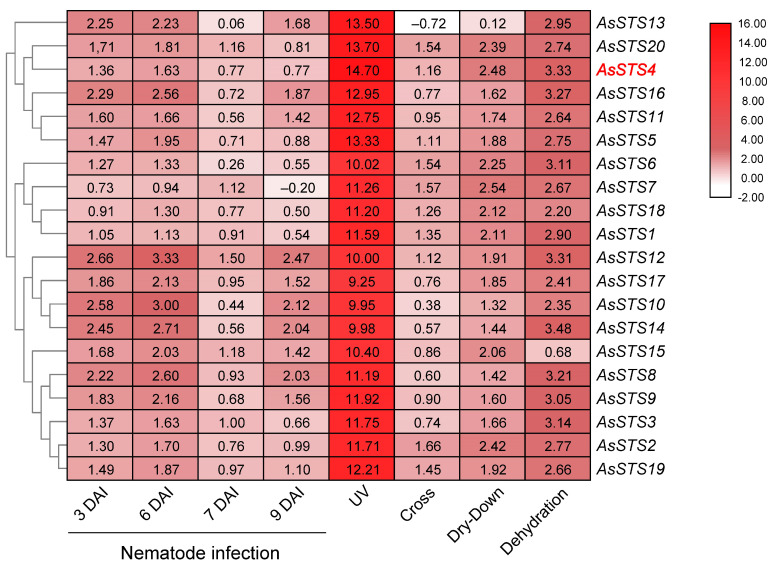
Heatmap of the in silico expression patterns of 20 *Arachis stenosperma* STS genes in response to different types of stresses: nematode infection (at 3, 6, 7, and 9 days after infection; DAI); ultraviolet (UV) exposure; drought treatments (dry-down and dehydration); and combined drought imposition and nematode infection (cross). The color key represents differential gene expression magnitude in Log2 fold change (FC) values.

Expression profiling showed that all *A. stenosperma* STS genes were upregulated in response to all isolated or combined stresses analyzed, with variations in their expression levels according to the treatment (Figure 9). The exceptions were the *AsSTS13* and *AsSTS7* genes that were downregulated in response to only one stress each: cross-stress and nematode infection at 9 DAI, respectively. Remarkably, a general strong induction by UV exposure (Log2 FC > 9.25) was observed for all *A. stenosperma* STS genes, exhibiting differences in expression at least eight times more than the other abiotic and biotic stresses (Figure 9). Across the 20 A. *stenosperma* genes, *AsSTS4* was the one that exhibited the most significant induction (Log2 FC = 14.7) upon UV stress and among those that displayed the highest levels of expression in response to all other applied stresses. (Figure 9). Although it is well-known that controlled UV radiation can lead to increased plant resistance to pathogens by inducing specialized metabolites and hormones, antioxidative activities, and defensive responses [37], the molecular mechanisms behind the general induction of wild *Arachis* STS genes following UV exposure or in response to different types of stresses are still not completely understood.

On the other hand, the transcript levels of the five *A. stenosperma* CHS genes were slightly affected by abiotic and biotic stresses analyzed, showing general magnitudes of expression much lower than those observed for STS genes (Figure S3). *AsCHS5* was the only gene significantly induced by dehydration and repressed upon UV stress or during the earlier stages (3 and 6 DAI) of nematode infection.

Further analysis showed that the number of transcriptional regulatory elements in the promoter region varied among the 20 *A. stenosperma* STS genes, ranging from 15 to 37 (Figure S4). We observed that the six genes forming the group with the highest expression abundance across all stresses (*AsSTS4*, *5*, *11*, *13*, *16*, and *20*; Figure 9) have different types and numbers of *cis*-elements in their promoters (Figure S4). The exception was *AsSTS4*, which, besides exhibiting the most significant induction upon UV stress, also showed the highest number of *cis*-elements (33 in total) distributed in the three categories (LRE, HRE, and STRE) involved in plant stress responses, such as MYB, ABRE, STRE, MYC, and Box4 (Figure S4). Therefore, to better understand the role of wild *Arachis* STS genes in the molecular response underlying their general induction by different biotic and abiotic stresses, we selected the *AsSTS4* gene for further *in planta* functional studies.

### 2.7. Functional Characterization of AsSTS4 by Overexpression in Peanut Hairy Roots

**Figure 10:**
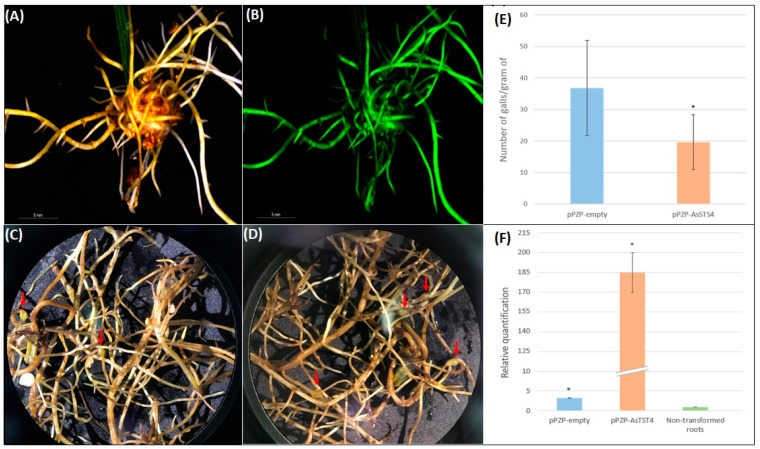
Peanut hairy roots transformed with *A. rhizogenes* harboring pPZP-AsSTS4 vector and observed under a stereomicroscope using bright field (**A**) and epifluorescence (**B**) 30 days after *A. rhizogenes* transformation. Transgenic peanut hairy roots transformed with pPZP- AsSTS4 (**C**) and pPZP-empty vectors (**D**), showing gall formation 60 days after *M. arenaria* inoculation. Mean number of galls per gram of transgenic hairy roots (**E**) and relative quantification of *AsSTS4* gene in transgenic peanut hairy roots transformed with pPZP-empty, pPZP-ASSTS4, and in non-transformed control roots (**F**). Arrows indicate nematode galls. Error bars mean the standard error of samples, and asterisks mean significant differences between samples (*p* < 0.05; Student’s *t*-test).

Roots emerging from the petiole-wounding site of peanut detached leaves 20 days after *A. rhizogenes* transformation were analyzed for GFP fluorescence. Most (90%) detached leaves transformed with pPZP-empty and pPZP-AsSTS4 vectors produced eGFP-positive hairy roots, with an average of six hairy roots per leaf. Only roots that displayed GFP fluorescence and typical hairy root phenotype were inoculated with nematodes (*M. arenaria*) approximately 30 days after *A. rhizogenes* transformation (Figure 10A,B).

The effect of *AsSTS4* overexpression in nematode infection was evaluated in the hairy roots at 60 days after inoculation by analyzing the root biomass and the number of galls. Hairy roots transformed with pPZP-AsSTS4 showed an average biomass of 0.29 ± 0.09 g per detached leaf that did not differ significantly (*p* < 0.05, *t*-test) from that observed in roots transformed with pPZP-empty vector (0.25 ± 0.16 g) (Figure 10C,D), suggesting that the induction and development of hairy roots were not affected by *AsSTS4* overexpression. Nematode infection was further confirmed in GFP-positive hairy roots derived from pPZP-empty vector transformation by gall development (average of 36.87 galls per root gram) (Figure 10C,E), corroborating previous studies demonstrate that *M. arenaria* could penetrate and develop inside RNK-susceptible peanut hairy roots [38,39,40]. In contrast, the overexpression of *AsSTS4* reduced the ability of *M. arenaria* to complete its life cycle in transgenic hairy roots (average of 19.65 galls per root gram) (Figure 10D,E), promoting a substantial and significant (*p* < 0.05, *t*-test) reduction of 46.71% in nematode infection in comparison to the control roots that did not express the transgene.

In addition, qRT-PCR analysis showed that *AsSTS4* overexpression in transgenic roots was 185 times higher than in the non-transformed controls (Figure 10F), confirming the transgenic status of the leaf-derived peanut hairy roots. We also observed the expression of an endogenous *AsSTS4* ortholog gene (3.2 X) in the hairy roots transformed with the pPZP-empty (Figure 10F), probably due to the intrinsic ability of peanut hairy roots to enhance resveratrol production, even under non-elicited conditions [41].

## 3. Discussion

Wild *Arachis* species evolved in their native South America several adaptive traits associated with defense and survival under stressful environmental conditions, among which the production and accumulation of phenolic-like compounds or phytoalexins seem to play a primary role [12,13,42]. Resveratrol is a major phytoalexin involved in constitutive and inducible defense reactions of *Arachis* species against bioaggressors, including fungi, bacteria, nematodes, and herbivores, and in response to several abiotic stressors, such as UV irradiation, wounding, drought, or extreme temperatures [10,42]. *Arachis* is one of the few plant genera that naturally produce resveratrol, and our recent findings demonstrate that this particularity is ubiquitously present in wild and cultivated species belonging to the different sections of the genus [12,13]. Thus, resveratrol and derivatives are important components of the defense mechanisms evolved by *Arachis* to cope with environmental constraints. Moreover, besides resveratrol, interest has been increasing in novel bioactive prenylated stilbenoids produced by both wild and cultivated *Arachis*, such as arachidins, and their potential medical benefits have been reported [16,41,42]. Here, the comprehensive characterization of CHS and STS genes in four *Arachis* species expands the molecular and evolutionary understanding of gene families involved in developing defensive secondary metabolites found during the environmental adaptation of wild species.

Previous studies have identified and characterized members of CHS and STS gene families in peanut (*A. hypogaea*), whereas there have been no reports on these families on wild *Arachis* species [43,44,45,46]. The allopolyploid domesticated peanut, with an AABB genome, arose from natural hybridization between the diploid ancestors *A. duranensis* (genome A) and *A. ipaënsis* (genome B), followed by spontaneous chromosome duplication [23]. *Arachis* genome evolution studies suggest that wild progenitors experienced one round of whole genome duplication, whereas the cultivated peanut experienced two rounds, with few changes in A- and B-subgenomes since polyploidization [24]. In the present study, the comprehensive characterization of CHS and STS gene families identified 52 members in *A. hypogaea*, whereas 27 and 26 members in *A. duranensis* and *A. ipaënsis*, respectively. As expected, the number of CHS (6) and STS (46) genes in the tetraploid *A. hypogaea* is almost equivalent to the combined value in its diploid wild progenitors, i.e., 8 CHS and 45 STS. Similar results have been reported for genome-wide analysis of the Phospholipase D gene family in *Arachis* [47] and for other gene families in *Brassica* and *Gossypium* allotetraploid species when compared with their corresponding diploid progenitors [48,49,50].

Analysis of the intron-exon organization of the 111 *Arachis* STS genes revealed a well-conserved intra- and interspecies structure in terms of both number and length of introns, which is consistent with the previously described structure for STS genes in plants [19,20]. The exception from the general 2-exon/1-intron organization of *Arachis* STS genes is *A. ipaënsis*, which showed dissimilar structures for some genes, such as intron absence or too big introns, which can be due to sequencing errors or genome annotation problems in this species. This highly conserved exon–intron architecture in both tetraploid and diploid genomes suggests that *A. hypogaea* STS genes did not undergo any mutation impact, either intron loss or intron gain during the polyploidization.

In addition to sharing very similar exon–intron arrangements, almost all *Arachis* STS genes (88%) are physically located in single aligned clusters in a single chromosome of each diploid species or the collinear chromosomes of the tetraploid species. This clustering within a limited region in a specific chromosome seems to be a particular feature of plant STS genes and was previously reported for grapevine and mulberry, two other resveratrol-producing plants for which a comprehensive analysis of the STS family was conducted [7,18,19,20]. In accordance with these studies, we found that the *Arachis* STS gene clusters are characterized by the presence of STS pseudogenes and other non-STS functional genes along with functional STS genes and enriched for TE copies, including retrotransposons and DNA transposons. TEs play a predominant role in cluster formation by chromosomal rearrangements, and their occurrence throughout the STS clusters can be associated with the frequency of recombination events in these regions. TE-rich regions are also often associated with genes involved in immunity or secondary metabolism in plants and are considered relevant compartments for phenotypic plasticity and adaptation to stress [51]. In addition, TEs are dynamic components of wild *Arachis* genomes, contributing very substantially as a primary driving force for the divergence of the A and B genomes [21,22,23,52]. In plants, genes involved in the biosynthesis of specialized metabolites are commonly clustered together with a coordinated expression to ensure sufficient production of metabolites and prevent the accumulation of toxic metabolic intermediates [53]. The presence of TEs facilitates the formation of these metabolic gene clusters. Moreover, as observed for *Arachis* STS, plant metabolic gene clusters comprise primarily the genes responsible for determining a class of metabolites with sizes ranging from 35 kb to several hundred kb [53]. However, to date, the phenomenon of plant metabolic gene clustering has not been associated with stilbenoids, with only two other gene clusters involved with the phenylpropanoid biosynthetic pathways so far being described [54].

Here, the clustered *Arachis* STS genes are also characterized by the presence of several common *cis*-acting elements in their promoter regions. As observed for the number of STS genes, ploidy level is the leading cause of *cis*-acting element number variation among *Arachis* species. In the category related to hormone response, *cis*-elements involved in ABA, ethylene, and MeJA signaling and biosynthesis (ABRE, ERE, and CGCTCA-, and TGACG-motif) were found with the highest frequency (72%) in the STS promoters of all *Arachis* species. In the category related to stress response, the most common (88%) *cis*-elements are those involved in plant responsiveness to drought, cold, salt, wounding, heat, and anaerobic conditions, such as STRE, MYB, ARE, W-box, and WUN-motif. Likewise, two ubiquitous regulatory elements associated with light-controlled transcriptional activities in plants (Box 4 and G-Box) are highly represented (58%) in the light-responsive category. The occurrence pattern of these well-recognized *cis*-elements involved in plant responsiveness to different environmental elicitors might reflect the importance of transcriptional regulation of STS genes and the resulting resveratrol accumulation in the adaptation and appropriate development of wild *Arachis* under stressful environmental conditions [55,56]. In particular, the promoter regions of the majority of STS genes contain the MBS (MYB-binding site) *cis*-elements that can be recognized by MYB transcription factors, which have been characterized as regulators of stilbene synthesis in grapevine [57]. Likewise, W-box elements (WRKY-binding sites) are also found in the promoter regions of some primary and specialized metabolism genes, including CHS and PAL [56]. Some of the most frequent elements found in this study (Box 4, ABRE, G-Box, ARE, and CGTCA- and TGACG-motif) were also identified in the promoter regions of four *A. hypogaea* STS genes, suggesting that they may be responsible for the regulation of STS expression during biotic and abiotic stress responses through MeJA and SA signaling [43].

We also observed that the clustered *A. stenosperma* STS genes displayed very similar gene expression behavior in response to each of the eight distinct biotic and abiotic stresses, both in terms of behavior and magnitude. In particular, they are strongly induced by UV exposure, which agrees with our previous studies showing that UV radiation drastically alters the expression of STS genes in various *Arachis* species [11,12,13,14]. This marked activation of STS gene expression in wild and cultivated *Arachis* is associated with increased resveratrol content after UV exposure. In particular, *A. stenosperma* is well-known to harbor high resistance levels against the RKN *M. arenaria* and multiple fungal diseases [58,59]. Therefore, as a critical phytoalexin selectively accumulated in response to biotic stress, enhanced accumulation of resveratrol could play a direct role in the broad-spectrum defense mechanisms developed by this wild species to withstand pathogen pressure.

In resveratrol-producing plants, CHS and STS share the same substrate to produce naringenin chalcone and resveratrol, respectively. Previous studies suggest that under stress conditions, these plants divert the common substrate to the resveratrol synthesis pathway over the naringenin chalcone synthesis in a competitive or inhibitory relationship [18,60,61]. Here, we also found that UV exposure strongly induced the expression of all 20 *A. stenosperma* STS genes, which are directly involved in resveratrol biosynthesis. In contrast, the five CHS genes are slightly responsive to UV. Likewise, STSs were upregulated by all the other biotic and abiotic stresses tested, whilst they did not significantly affect the expression of CHS genes. Notably, the number of STS members identified in the four *Arachis* species is four to eight times higher when compared to CHS members, regardless of their ploidy levels. This larger size of the STS family compared to CHS was also reported in other resveratrol-producing plants, with grapevine and mulberry showing 3.5 and 1.6 more STS than CHS complete genes, respectively [19,20]. Conversely, in non-producing resveratrol eudicots plants, CHS form large gene families, such as in soybean and mango (both with 21 members) and cotton (20 members), whilst no STS genes have been identified [27,28,29]. In accordance with [43], our phylogenetic analysis confirms the independent evolution of STS genes in *Arachis* species from 11 non-producing resveratrol legumes. Therefore, as proposed for grapevine and *Polygonum* [18,60,61], our findings reinforce the existence of antagonism between flavonol and stilbene biosynthesis, which prioritizes resveratrol accumulation under stress conditions and is likely to play an important role in the adaptation of wild *Arachis* to stressful environments [6].

Based on the highly conserved gene structure, the presence of common regulatory elements, and the very similar gene expression behavior in response to distinct types of stress, functional conservation of *Arachis* STS corresponding proteins might be predicted among the different *Arachis* species. Indeed, all *Arachis* STS share extensive amino acid sequence identity, with the conserved motifs 1, 3, and 4 representing the Chal_sti_synt_N domain and motifs 2 and 5 comprising the Chal_sti_synt_C domain present in all CHS and STS proteins, suggesting conserved evolution. Moreover, they show absolute conservation of the internal active site, including the Cys-His-Asn catalytic triad inherited from the KAS III ancestor, which is considered a very important feature for the catalytic function of CHS and STS enzymes in plants [1]. The phylogenetic relationships of *Arachis* proteins also showed that the CHS clustered separately, as outgroups, from all STS. Both CHS and STS clades formed subclades according to the chromosome position of their corresponding genes. The existence of a unique clade composed exclusively of STS suggests a conservation of the biological function amongst all *Arachis* STS proteins.

STS are stress-inducible downstream genes, and the consequent accumulation of resveratrol seems to be an important component of *Arachis* mechanisms underlying defense responses against biotic and abiotic stresses. In order to shed light on the role of wild *Arachis* STS genes in stress responses, we selected the *A. stenosperma AsSTS4* gene as the candidate for a further functional assignment. Besides being highly responsive to different types of stresses, *AsSTS4* presented the highest number of *cis*-regulatory elements involved in sensing environmental signals. The *in planta* validation of biological functions of candidate genes is a critical step to reveal its usefulness in the future for genetic breeding programs towards resistance to environmental stresses. Therefore, *AsSTS4* was overexpressed in transgenic hairy roots of an RKN-susceptible peanut genotype, and its effect on RKN infection was evaluated. Our results showed that *AsSTS4* significantly improved resistance against *M. arenaria*, one of the most damaging phytoparasites to peanut yield worldwide, causing significant crop loss and expensive control measures [62].

Previous studies suggested that resveratrol, besides its classical role as antimicrobial phytoalexin, constitutes an important regulator for the initiation of a hypersensitive response (HR) cell death, as described for *A. stenosperma* against *M. arenaria* [58,63,64]. Overexpression of STS genes isolated from cultivated peanut has already been reported in transgenic sweet potato and rice, both aiming to produce resveratrol in heterologous systems [17,45,46]. To our knowledge, the present study is the first report that applies the heterologous overexpression of an STS gene to increase nematode resistance, providing new functional insights into its role in biotic stress responses. As wild *Arachis* is one of the few plant genera that naturally synthesize resveratrol, our findings offer new perspectives for the biotechnological exploitation of these wild STS genes to increase plant resistance to biotic and abiotic stresses along with their pharmaceutical, cosmetic, and industrial purposes.

## 4. Conclusions

In the present study, peanut and its wild relatives join grapevine and mulberry as resveratrol-producing plants for which a comprehensive analysis of the CHS and STS gene families has been conducted. A total of 52 members were identified in allopolyploid domesticated peanut, while half were found in its wild diploid relatives *A. duranensis*, *A. stenosperma*, and *A. ipaënsis* (27, 25, and 26 members, respectively). This comprehensive analysis revealed strong conservation of these *Arachis* genes regarding gene and protein structures and the presence of common regulatory elements involved in stress signaling responses. In particular, the clustering of *Arachis* STS genes in one chromosome and their coordinated response to stress suggest a functional role for this physical organization, such as the plant metabolic gene clustering phenomenon, which has not yet been described for plant metabolic genes involved with the phenylpropanoid biosynthetic pathways. The general induction of *Arachis* STS genes in response to biotic and abiotic stress conditions, not observed for the CHS genes, corroborates the existence of an antagonism between these competitive biosynthesis pathways that prioritizes induction of STS genes under stressful conditions. Moreover, the overexpression of the wild *AsSTS4* gene enhanced the resistance of peanut roots to RKN, which might be further exploited for biotechnological purposes. Overall, these findings support the idea that *Arachis* STS family members and resveratrol accumulation play an important role in adapting wild *Arachis* to stressful environments, increasing our understanding of the constitutive and inducible defense reactions mediated by this relevant secondary metabolite.

## 5. Materials and Methods

### 5.1. Identification of CHS and STS Gene Families in Four Arachis Species

Two steps were used to identify CHS and STS gene family members in *Arachis* spp. First, a gene family search in the PeanutBase database (http://peanutbase.org/; accessed on 15 May 2023) was performed using the Prosite entry PS00441 equivalent to CHS/STS active site (https://prosite.expasy.org/, accessed on 5 May 2023), and also the InterPro entry IPR011141, corresponding to type III PKSs family domains (https://www.ebi.ac.uk/interpro/, accessed on 15 May 2023). The search was conducted in the annotated genomes of cultivated peanut (*A. hypogaea* Tifrunner v. 1.0) and two wild *Arachis* species (*A. duranensis* V14167 v.1.0 and *A. ipaënsis* K30076 v. 1.0). Next, each putative CHS and STS genemodel sequence was used as a query for nucleotide BLAST (BLASTn) search against the genomic sequences of *A. hypogaea*, *A. duranensis*, *A. ipaënsis*, and *A. stenosperma* from both NCBI (https://www.ncbi.nlm.nih.gov/, accessed on 10 June 2023) and PeanutBase (http://peanutbase.org/, accessed on 10 June 2023). Non-redundant CHS and STS genes were selected. The molecular weight and other physicochemical properties were predicted for inferred CHS and STS protein sequences using the web-based tool ExPASy (https://web.expasy.org/, accessed on 21 July 2023). The CHS and STS protein sequences were used as input for the Plant-mSubP webserver (http://bioinfo.usu.edu/Plant-mSubP/, accessed on 30 July 2023) to predict their subcellular localization. All databases and tools were used with default settings.

### 5.2. Gene Localization and Synteny Analysis of Arachis STS/CHS Genes

The MCScanX toolkit [65] was used to search for duplication and synteny in a cross-species manner for three species (*A. duranensis*, *A. ipaënsis*, and *A. hypogaea*), using the collinear relations to plot the synteny graph. The output from McScanX was formatted using an in-house script (https://github.com/lbi-cenargen, accessed on 10 August 2023) to retrieve all syntenic genes from CHS and STS gene families and produce an input file for the circa visualization software v1.0 (OMGenomics; http://omgenomics.com/circa/, accessed on 20 August 2023).

The physical organization of the STS clusters on chromosomes 04 and 14 of *Arachis* spp. was predicted using the GBrowse tool from PeanutBase (http://peanutbase.org/, accessed on 10 July 2023), accessed on 21 July 2023. To search putative transposable elements (TEs), the mixed repeats sequences from *A. ipaënsis*, *A. duranensis*, and *A*. *hypogaea* identified by [21] were used as a query for BLASTn search against the genome sequence of each STS cluster. The distribution and orientation of STS, CHS, TEs, STS pseudogenes, and other functional genes within each cluster were then arranged using SnapGene^®^ software v5.0.8 (https://www.snapgene.com, accessed on 25 July 2023).

### 5.3. Arachis CHS and STS Gene and Protein Structures

The exon–intron organization of *Arachis* CHS and STS genes retrieved from PeanutBase (http://peanutbase.org/, accessed on 10 June 2023) was represented using GSDS software v2.0 (http://gsds.gao-lab.org, accessed on 21 June 2023). Protein sequences were submitted to the web-server based tool DomainViz (https://uhrigprotools.biology.ualberta.ca/, accessed on 15 June 2023) to visualize the distribution and organization of conserved PFAM domains. The online tool MEME Suite (https://meme-suite.org/meme/, accessed on 25 June 2023) was used to predict conserved motifs in *Arachis* CHS and STS protein sequences, with the motif length ranging from 6 to 100 residues and maximum number of motifs to 10. A phylogenetic tree was constructed using MEGA 11 software v11.0.13 [66] and then visualized with protein motifs via TBtools-II software v2.0 [67].

### 5.4. Phylogenetic Analysis of CHS and STS Members in Arachis spp.

The amino acid sequences predicted from the STS and CHS gene sequences of *A. duranensis*, *A. ipaënsis*, *A. stenosperma*, and *A. hypogaea* were aligned and used to obtain a phylogenetic tree using MEGA 11 software [66]. The sequence alignment, obtained using Clustal W, was used to infer evolutionary history using the Maximum Parsimony method that yielded a 1000 replicates bootstrap consensus tree.

### 5.5. Analysis of Cis-Elements of CHS and STS Upstream Sequences

For the analysis of *cis*-acting regulatory elements, the sequence of 1500 nucleotides upstream of the translation initiation codon of each CHS and STS gene from *A. duranensis*, *A. ipaënsis*, *A. stenosperma*, and *A. hypogaea* was retrieved from Phytozome (https://phytozome-next.jgi.doe.gov/, accessed on 30 August 2023) and PeanutBase (https://peanutbase.org/home, accessed on 30 August 2023) databases.

The extracted sequences of promoter regions were submitted to the PlantCARE database (http://bioinformatics.psb.ugent.be/webtools/plantcare/html/, accessed on 17 September 2023 [68] for the prediction of *cis*-acting elements. All results were manually mined, and resultant hits of different cis-regulatory elements in each *Arachis* CHS and STS gene were visualized using TBtools-II [67].

### 5.6. Expression Analysis of CHS and STS Genes in Response to Biotic and Abiotic Stresses

The expression profiles of *A. stenosperma* STS genes were subjected to heatmap construction by TBtools-II software 2.0 [67] using our previously produced transcriptome RNA-Seq data from plants submitted to different types of stresses: *M. arenaria* infection (at 3, 6, 7 and 9 DAI); UV exposure; moderate (dry-down) and severe (dehydration) drought treatments; and combined drought and nematode stresses (cross-stress) [36,63,69,70].

### 5.7. Overexpression of AsSTS4 Gene in Peanut Hairy Roots

The predicted coding region (1173 bp; Table S3) of *AsSTS4* from *A. stenosperma* was synthesized and cloned, under the control of the *Arabidopsis thaliana* actin 2 promoter, into the unique *XhoI* restriction site of the binary vector pPZP-BAR [70] by Epoch Life Science (Missouri City, TX, USA). The obtained vector pPZP-AsSTS4 and the corresponding empty pPZP-BAR vector, hereafter called pPZP-empty, were then introduced into the wild cucumopine-type *Agrobacterium rhizogenes* strain ‘K599’ and grown on selective Luria–Bertani (LB) medium to produce a fresh bacterial paste inoculum, essentially as described by [39].

Hairy roots of the RKN-susceptible peanut ‘Runner IAC-866’ were produced by the ex vitro detached leaf method previously established by our group [38,39], using quadrifoliate leaves harvested from one-month-old peanut plants grown under growth chamber conditions (25 ± 2 °C; 12 h photoperiod; 120 µmols m^−2^ s^−1^ light intensity). Detached leaves containing only eGFP-positive hairy roots were selected approximately 20 days after *A. rhizogenes* transformation and covered with medium-grained (5 to 8 mm) vermiculite for nematode infection.

### 5.8. Hairy Roots Inoculation with Meloidogyne arenaria

*M. arenaria* were multiplied for three months on greenhouse-grown tomato (*Solanum lycopersicum* ‘Santa Clara’) plants and second-stage juveniles (J2) collected essentially as described by [40]. The identity of *M. arenaria* inoculum was confirmed by PCR analysis using a specific SCAR marker [71], as previously established [72]. GFP-positive hairy roots transformed with pPZP-AsSTS4 and pPZP-empty vectors were then challenged with approximately 1000 J2 of *M. arenaria* according to [38] and maintained in growth chamber conditions.

At 60 DAI, hairy roots were carefully removed from the vermiculite, washed in running water, and weighted. The nematode infection in hairy roots transformed with both pPZP-AsAsSTS4 and pPZP-empty vectors was assessed by counting the number of galls under a stereomicroscope (Stemi 508, Zeiss, Oberkochen, Germany) and statistically analyzed using Student’s *t*-test (*p* < 0.05).

### 5.9. Transgene Expression Profiling

qRT-PCR analysis of *AsSTS4* expression in peanut hairy roots was evaluated using specific primer pairs (5′-3′) STS1 (AAGGCCATCAAGGAATGGGG/ATGGGTCGAGGCCTAAGAGT) [36]. Total RNA was extracted from hairy roots in biological triplicates using the RNeasy Plant Mini kit (Qiagen, Hilden, Germany) following the manufacturer’s instructions. As a negative control, total RNA was extracted from non-transformed roots of two-week-old peanut plantlets grown under growth chamber conditions. RNA purification, assessment of the RNA integrity, and cDNA synthesis were performed according to [73]. qRT-PCR reactions were conducted on a StepOne Plus Real-Time PCR System (Applied Biosystems, Foster City, USA), as described by [72]. Primer efficiency was determined by the online real-time PCR Miner tool [74], and the quantification of *AsSTS4* expression was estimated using the SATqPCR website [75]. *Arachis* GAPDH and 60S reference genes were used to normalize *AsSTS4* expression in accordance with [76].

## Figures and Tables

**Table 1:** Distribution of CHS and STS gene families in *Arachis duranensis* (2n), *A. ipaënsis* (2n), *A. stenosperma* (2n), and *A. hypogaea* (4n).

Species	Total of CHS- and STS-Coding Putative Proteins	Potential Pseudogenes *	Complete CHS and STS Genes			STS Gene Clusters		
			CHS Genes	STS Genes	Total	Chromossome **	Number of STS Genes	Density (Gene/100 kb)
*Arachis duranensis*	37	10	5	22	27	04	21 over 695 kb	3022
*Arachis ipaënsis*	38	12	3	23	26	04	18 over 922 kb	1952
*Arachis stenosperma*	36	11	5	20	25	04	17 over 817 kb	2082
Peanut (*Arachis hypogaea*)	74	22	6	46	52	04	16 over 622 kb	2571
						14	23 over 943 kb	2441

* Pseudogenes sequences contained either premature stop codons, frameshift mutations, or partial CHS/STS domains. ** In *Arachis hypogaea*, chromosomes 04 (A-subgenome) and 14 (B-subgenome) are collinear chromosomes.

**Table 2:** Total and mean number of *cis*-acting elements on STS and CHS gene promoters of *Arachis duranensis*, *A. ipaënsis*, *A. stenosperma*, and *A. hypogaea*.

Functional Categories *		HRE		LRE		STRE		TS&DEV		Total	
Species	Number of *cis*-Acting Elements	STS	CHS	STS	CHS	STS	CHS	STS	CHS	STS	CHS
*Arachis duranensis*	Total	108	30	149	30	114	40	81	14	452	114
	Mean	7.6	7.3	9.3	7.5	7.1	10	5.1	3.5	-	-
*Arachis ipaënsis*	Total	108	20	96	4	83	0	53	0	340	24
	Mean	10.8	**	9.6	**	8.3	**	5.3	**	-	-
*Arachis stenosperma*	Total	169	46	173	57	151	44	125	22	618	169
	Mean	8.5	8.6	8.7	11.4	7.6	8.8	6.3	4.4	-	-
Peanut (*Arachis hypogaea*)	Total	387	60	460	70	358	52	261	45	1466	227
	Mean	8.4	7.5	10	7.4	7.8	7.4	5.6	6.4	-	-

* HRE = hormone-responsive; LRE = light-responsive; STRE = stress-responsive; TS&DEV = tissue-specific and development. ** Only one *A. ipaënsis* CHS gene had the 1500 nucleotides promoter sequence available.

## Data Availability

Genome references for the genome and gene annotation data were downloaded from the following NCBI GenBank assembly accessions: *Arachis hypogaea* (GCF_003086295.2); *Arachis duranensis* (GCF_000817695.3); *Arachis ipaënsis* (GCF_000816755.2); and *Arachis stenosperma* (GCF_014773155.1).

## Supplementary Materials

The following supporting information can be downloaded at: https://www.mdpi.com/article/10.3390/genes14122181/s1, Table S1: Members of CHS and STS families in *Arachis duranensis* genome. Table S2: Members of CHS and STS families in *Arachis ipaënsis* genome. Table S3: Members of CHS and STS families in *Arachis stenosperma* genome. Table S4. Members of CHS and STS families in *Arachis hypogaea* genome. Figure S1: Gene tree of *Arachis* CHS and STS syntenic genes of three *Arachis* species (*A. duranensis*, *A. ipaënsis*, and *A. hypogaea*), 11 legumes and four non-legume species. Figure S2: Alignment of CHS and STS amino acid sequences of (A) *Arachis duranensis*; (B) *A. ipaënsis*; (C) *A. stenosperma*; and (D) *A. hypogaea*. (1) = STS and CHS specific residues around Met98; (2) = STS and CHS specific residues around Thr132; (3) = CHS/STS active site (Prosite entry PS00441); (4) = CHS/STS signature motif. Figure S3: Heatmap of the in silico expression patterns of five *Arachis stenosperma* CHS genes in response to different types of stresses: nematode infection (at 3, 6, 7, and 9 days after infection; DAI); Ultraviolet (UV) exposure; drought treatments (dry-Down and dehydration); and combined drought imposition and nematode infection (cross). The color key represents differential gene expression magnitude in Log2 fold change (FC) values. Figure S4: Heatmap of *cis*-acting elements (A) and their corresponding categories (B) in the promoter regions of 20 *Arachis stenosperma* STS genes. The heatmap colors ranged from red to blue, where red dark indicated increasing values in the numbers of *cis*-elements and blue scale decreasing values. *Cis*-acting elements are distributed in categories associated with responses to hormones (HRE), light (LRE), and stress (STRE) and related to tissue specificity and development (TS&DE).

## Abbreviations

CHS = Chalcone Synthase;
DAI = Days after Inoculation;
FC = Fold-Change;
HR = Hypersensitive Response;
PKS = Polyketide Synthases
qRT-PCR = quantitative Reverse Transcription-Polymerase Chain Reaction;
RKN = Root-Knot Nematode;
STS = Stilbene Synthase;
TE = Transposable Element;
UV = Ultraviolet.
