# Supplementary material for: The Stilbene Synthase Family in Arachis: A Genome-Wide Study and Functional Characterization in Response to Stress
Source: Genes (Basel). 2023 Dec 5;14(12):2181. doi: 10.3390/genes14122181 (PMC10742623; doi:10.3390/genes14122181)
Supplement: Supplementary file 1 [file genes-14-02181-s001.zip › genes-2718983-supplementary.pdf]

Supplementary files

The Stilbene Synthase Family in *Arachis*: A Genome-Wide Study and Functional Characterization in Response to Stress

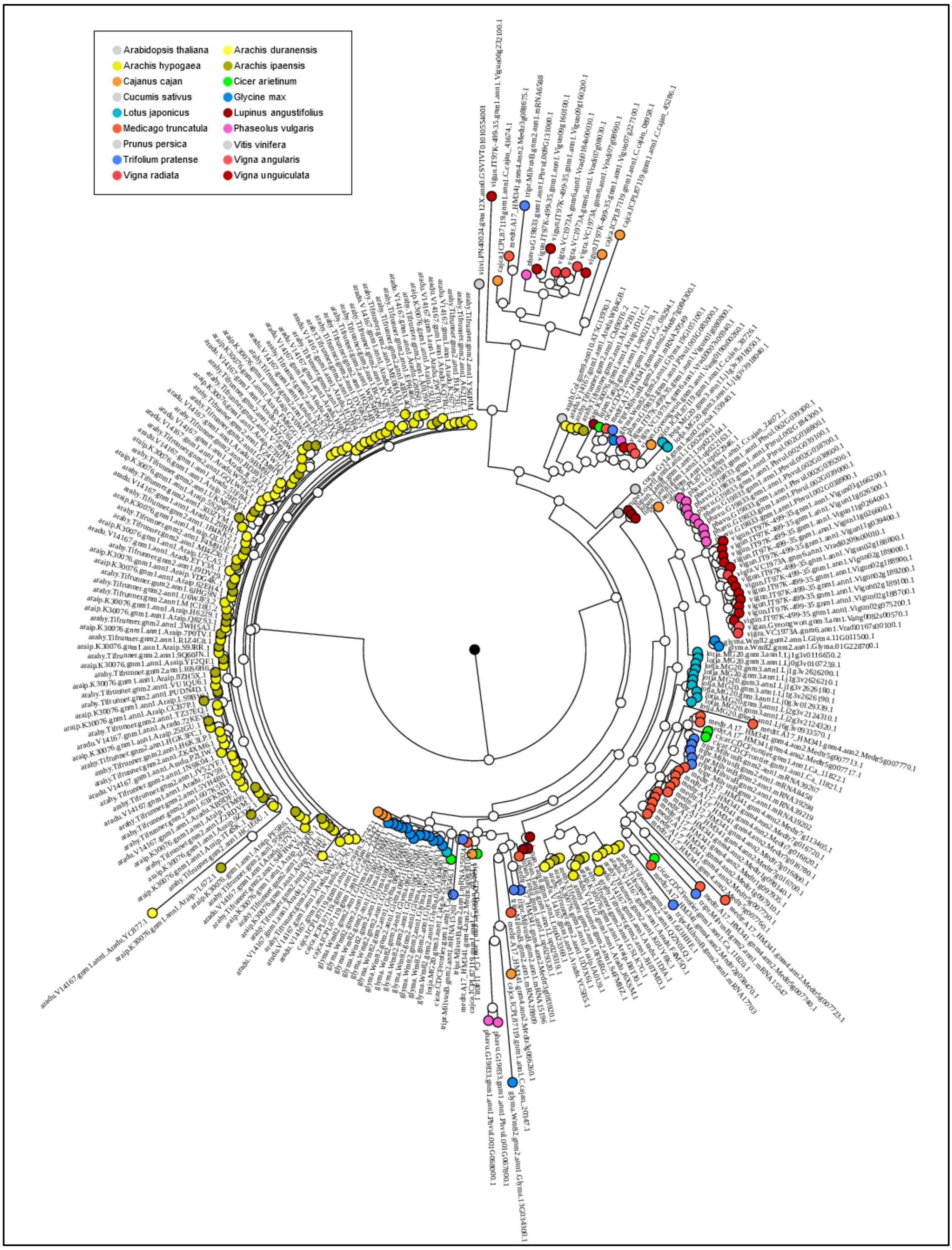

Figure S1. Gene tree of *Arachis* CHS and STS syntenic genes of three *Arachis* (*A. duranensis*, *A. ipaënsis*, and *A. hypogaea*), 11 legumes and four non-legume species.

(A)
Arachis duranensis

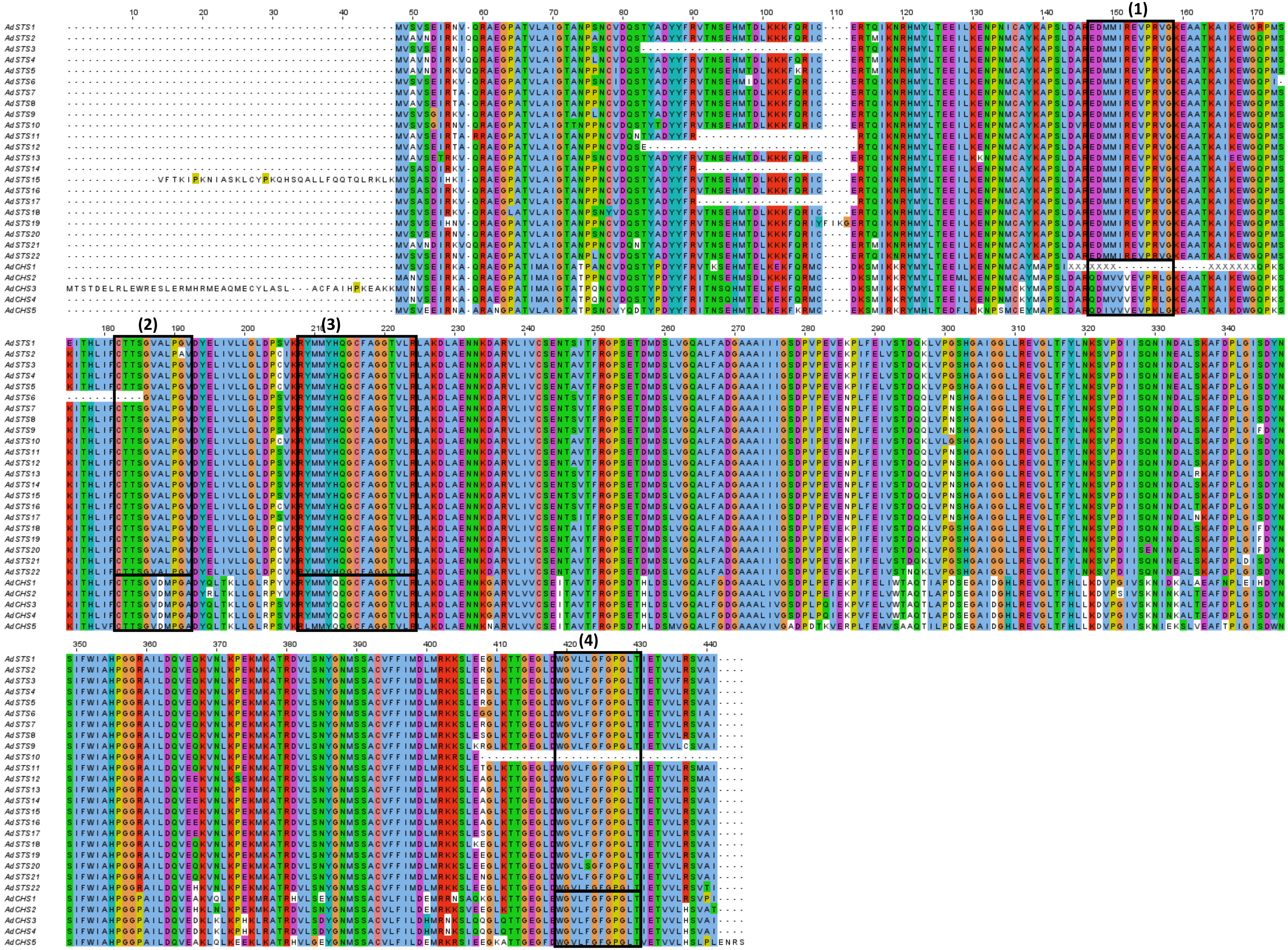

(B) *Arachis ipaënsis*

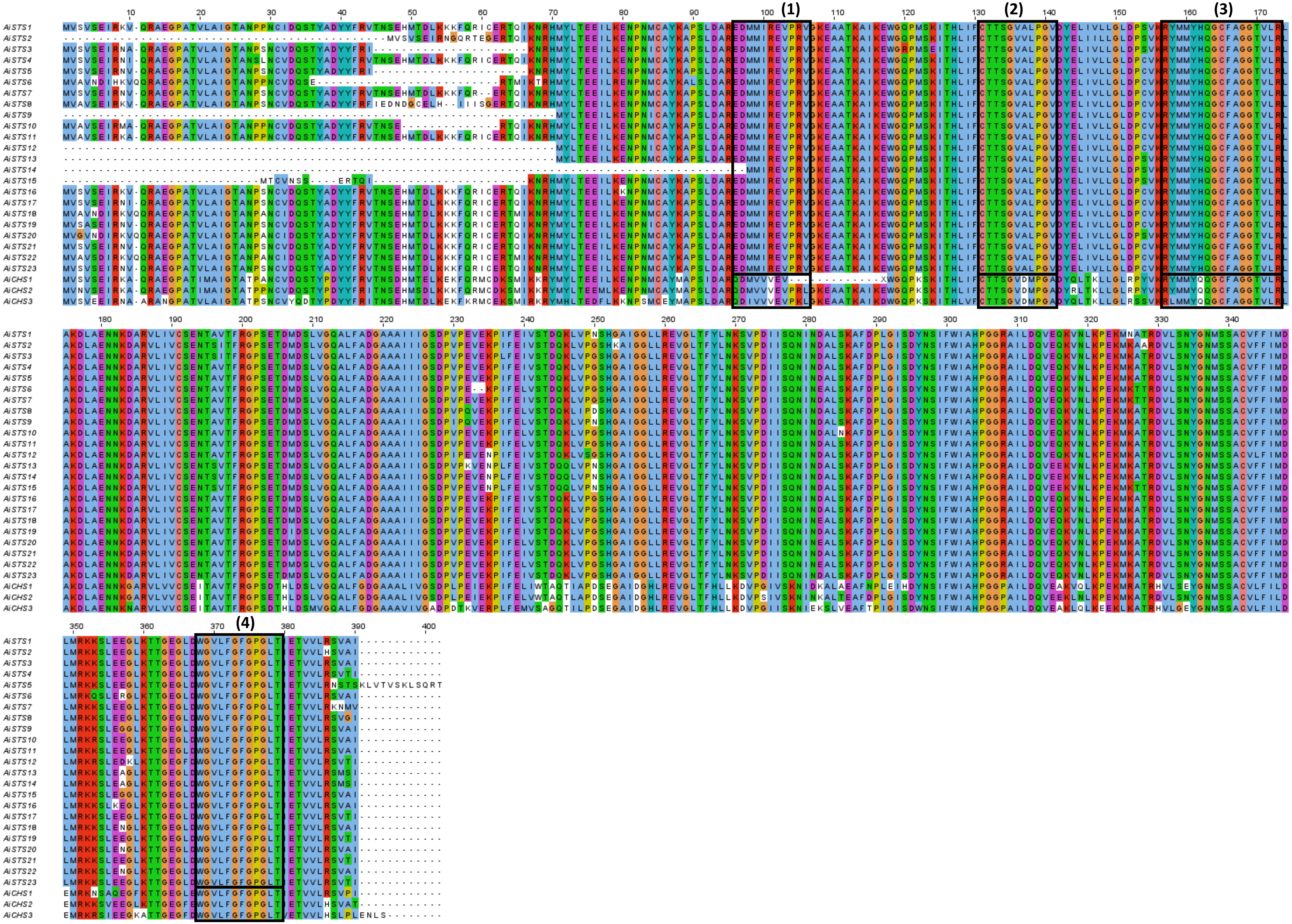

(C)
Arachis stenosperma

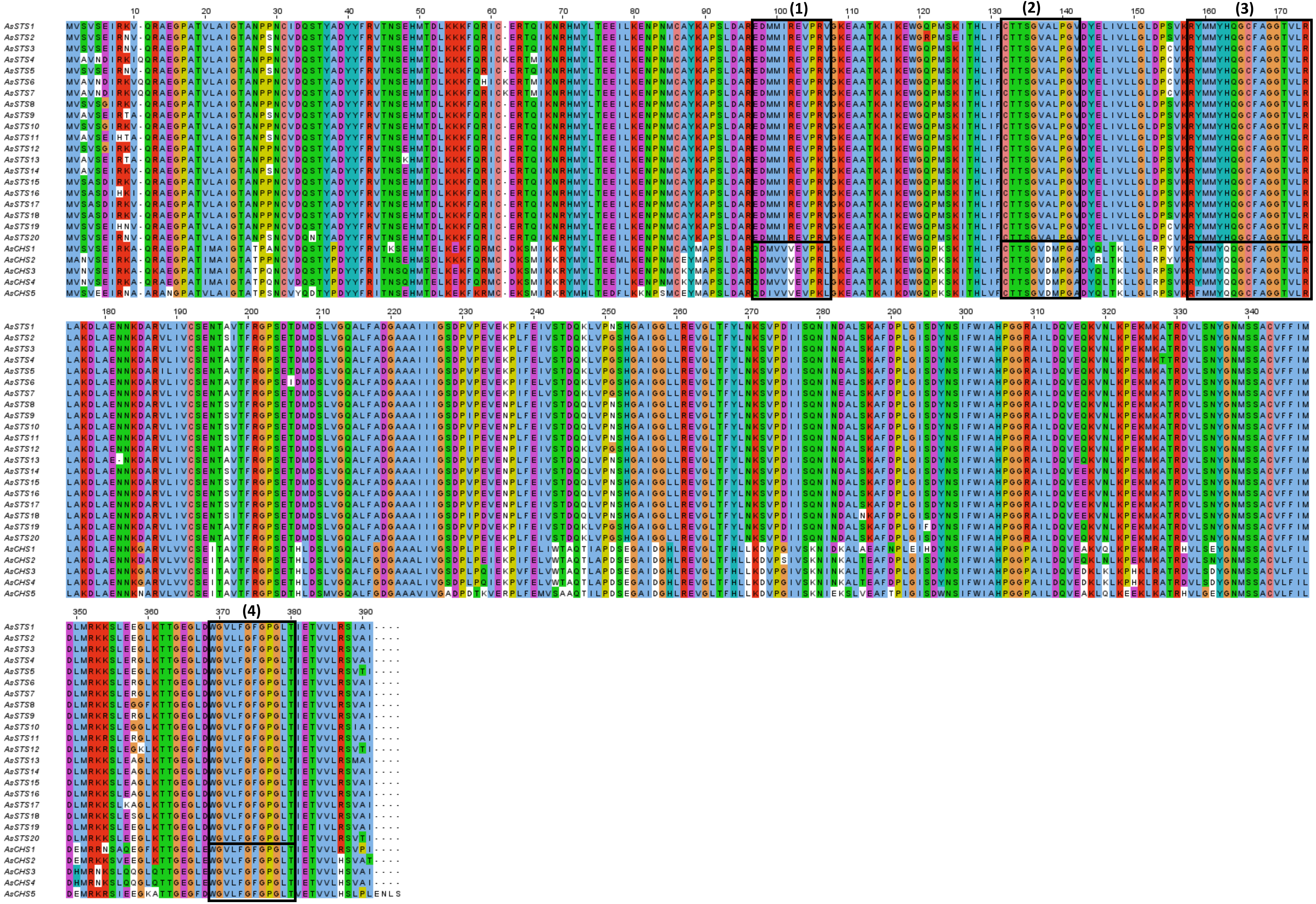

(D)
Arachis hypogaea

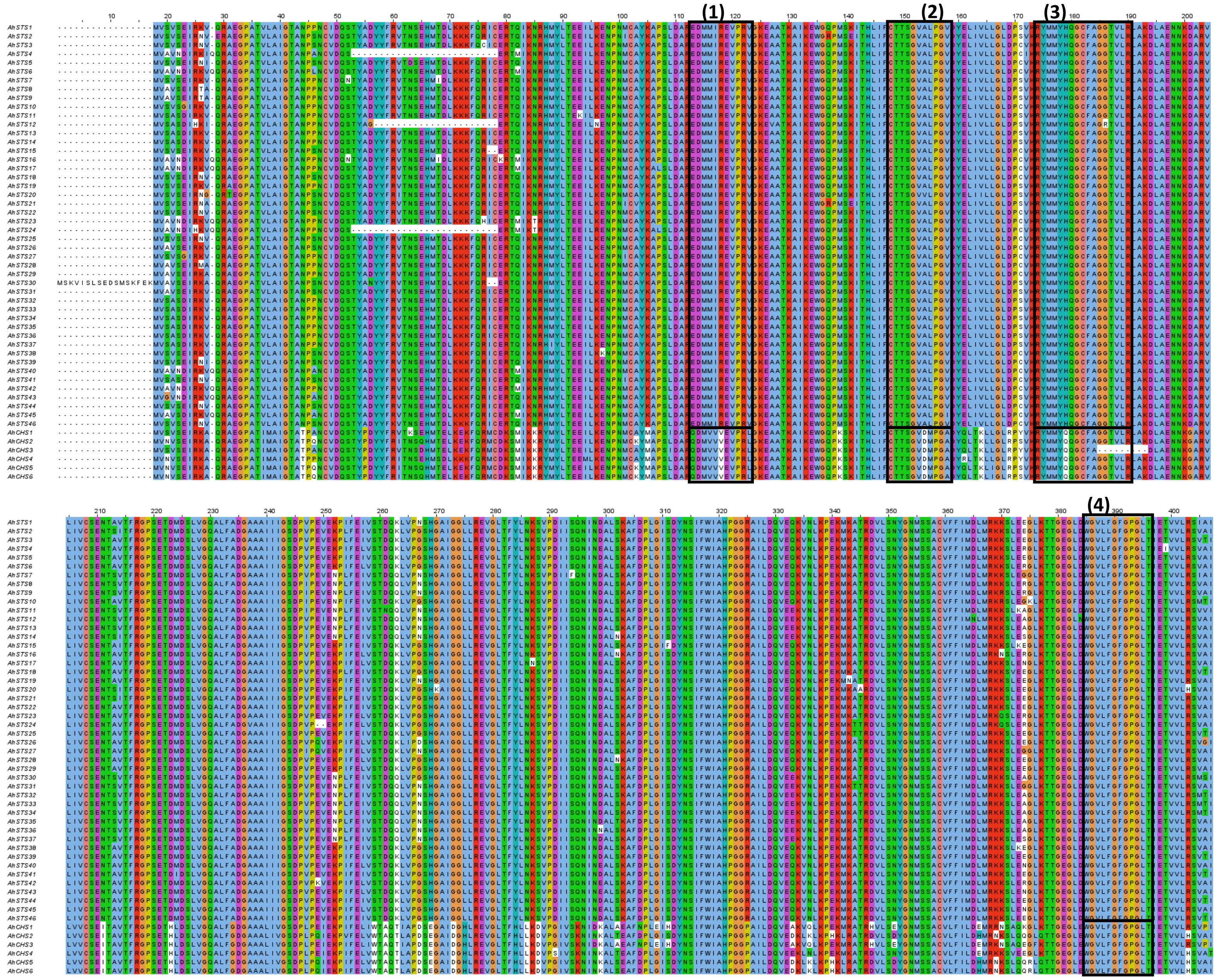

Figure S2. Alignment of CHS and STS amino acid sequences of **(A)** *Arachis duranensis*; **(B)** *A. ipaënsis*; **(C)** *A. stenosperma*; and **(D)** *A. hypogaea*. (1) = STS and CHS specific residues around Met98; (2) = STS and CHS specific residues around Thr132; (3) = CHS/STS active site (Prosite entry PS00441); (4) = CHS/STS signature motif.

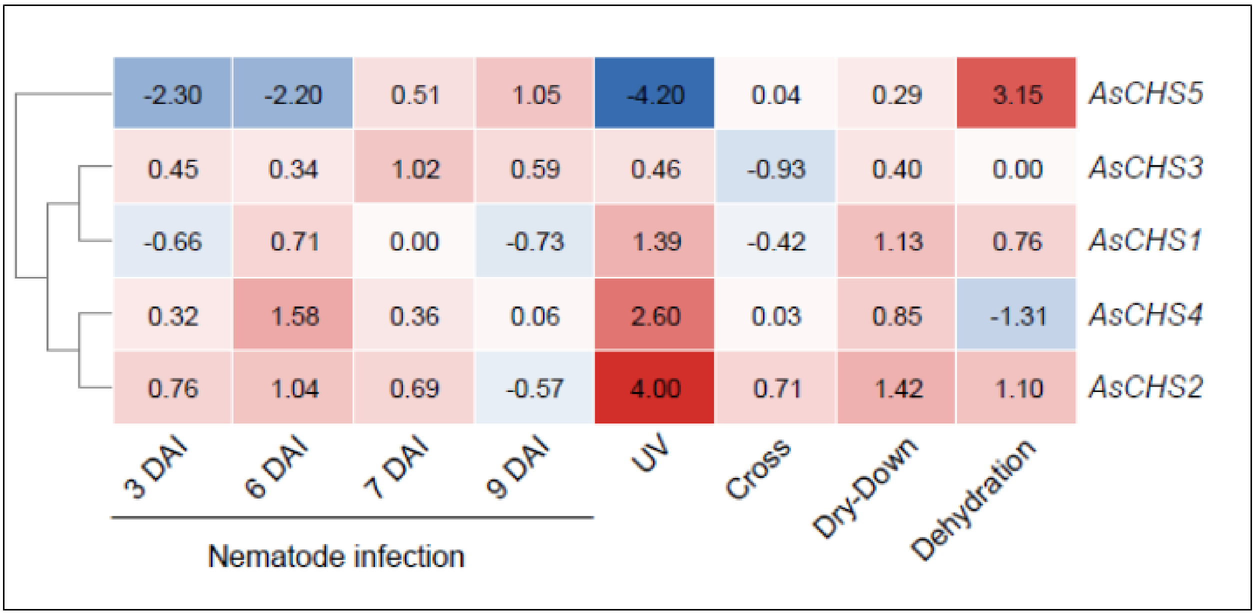

Figure S3. Heatmap of the *in silico* expression patterns of five *Arachis stenosperma* CHS genes in response to different types of stresses: nematode infection (at 3, 6, 7, and 9 days after infection; DAI); Ultraviolet (UV) exposure; drought treatments (Dry-Down and Dehydration); and combined drought imposition and nematode infection (Cross). The color key represents differential gene expression magnitude in Log2 fold change (FC) values.

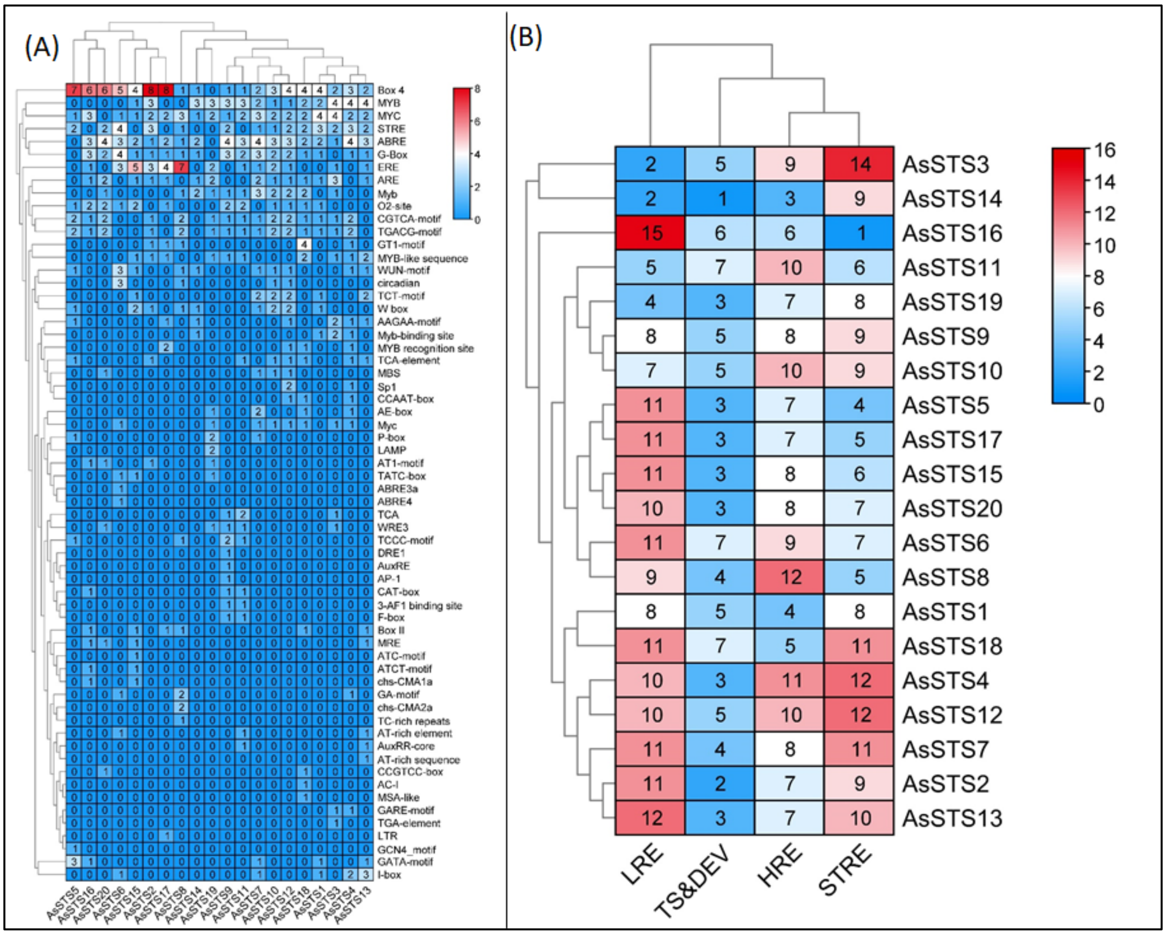

Figure S4. Heatmap of *cis*-acting elements (A) and their corresponding categories (B) in the promoter regions of 20 *Arachis stenosperma* STS genes. The heatmap colors ranged from red to blue, where red dark indicated increasing values in the numbers of *cis*-elements and blue scale decreasing values. *Cis*-acting elements are distributed in categories associated with responses to hormones (HRE), light (LRE) and stress (STRE) and related to tissue specificity and development (TS&DE).

Table S1. Members of CHS and STS families in *Arachis duranensis* genome.

Gene						Deduced Protein						Subcellular Localization (Plant-mSubP)
Nº	Nomenclature	PeanutBase ID*	Chromossome	Genome position*	ORF length (bp)	Size (aa)	MW (kDa)	pI	II	AI	GRAVY	
1	*AdCHS1*	Aradu.YC5B5	A03	Aradu.A03:8292185..8294622	2,437	376	40.00	6.47	38.79	85.61	-0.081	Cytoplasm
2	*AdSTS1*	Aradu.51F84	A04	Aradu.A04:1877892..1879665	1,773	389	42.79	5.67	36.81	93.98	-0.080	Plastid
3	*AdSTS2*	Aradu.XB9DF	A04	Aradu.A04:10419781..10421746	1,965	390	43.00	6.35	31.69	93.03	-0.059	Plastid
4	*AdSTS3*	Aradu.LZ0RH	A04	Aradu.A04:10471622..10473114	1,492	363	39.60	5.43	36.14	94.55	-0.013	Plastid
5	*AdSTS4*	Aradu.72V59	A04	Aradu.A04:10477696..10479657	1,961	390	43.00	6.35	30.66	93.00	-0.061	Cytoplasm
6	*AdSTS5*	Aradu.72KEV	A04	Aradu.A04:10494783..10496775	1,992	390	43.00	6.62	32.18	92.26	-0.075	Cytoplasm
7	*AdSTS6*	Aradu.P9QYX	A04	Aradu.A04:10517219..10518802	1,583	377	41.67	5.56	33.87	94.14	-0.088	Plastid
8	*AdSTS7*	Aradu.K2677	A04	Aradu.A04:10523116..10525012	1,896	389	42,83	5.79	32.65	91.75	-0.091	Plastid
9	*AdSTS8*	Aradu.KQ7RG	A04	Aradu.A04:10548950..10550856	1,906	389	42.76	5.65	33.37	91.75	-0.081	Plastid
10	*AdSTS9*	Aradu.537VV	A04	Aradu.A04:10560818..10562728	1,910	389	42.84	6.34	31.11	92.72	-0.052	Plastid
11	*AdSTS10*	Aradu.XCU6I	A04	Aradu.A04:10623792..10625303	1,511	355	39.35	6.12	30.82	90.90	-0.123	Plastid
12	*AdSTS11*	Aradu.W0CCT	A04	Aradu.A04:10645838..10647566	1,728	370	40.54	5.53	37.57	92.78	-0.043	Plastid
13	*AdSTS12*	Aradu.56C76	A04	Aradu.A04:10716985..10718721	1,736	363	39.49	5.22	37.03	94.57	-0.012	Plastid
14	*AdSTS13*	Aradu.ZWT01	A04	Aradu.A04:10723334..10725328	1,994	389	42.81	5.80	36.22	91.23	-0.097	Golgi apparatus
15	*AdSTS14*	Aradu.H9IK5	A04	Aradu.A04:10764667..10766177	1,510	370	40.47	5.41	36.43	93.30	-0.043	Plastid
16	*AdSTS15*	Aradu.WV5HP	A04	Aradu.A04:10815930..10817950	2,020	423	46.74	7.18	35.05	91.99	-0.117	Plastid
17	*AdSTS16*	Aradu.H7DF4	A04	Aradu.A04:10846459..10848377	1,918	390	42.79	5.65	32.19	91.49	-0.082	Plastid
18	*AdSTS17*	Aradu.G80S0	A04	Aradu.A04:10968649..10970160	1,511	370	40.56	5.31	36.49	93.57	-0.05	Plastid
19	*AdCHS2*	Aradu.J6XSM	A04	Aradu.A04:11029030..11030449	1,419	389	42.86	6.47	37.91	87.51	-0.137	Cytoplasm
20	*AdSTS18*	Aradu.9BM9J	A04	Aradu.A04:11054255..11056291	2,036	389	42.96	6.35	32.09	93.24	-0.060	Plastid
21	*AdSTS19*	Aradu.W79GG	A04	Aradu.A04:11061220..11063011	1,791	393	43.34	5.88	32.80	92.04	-0.063	Plastid
22	*AdSTS20*	Aradu.JL5FQ	A04	Aradu.A04:11065139..11066915	1,776	389	42.81	5.67	33.91	92.24	-0.071	Plastid
23	*AdSTS21*	Aradu.P2LIW	A04	Aradu.A04:11109622..11111683	2,061	390	43.03	5.96	29.86	92.00	-0.087	Cytoplasm
24	*AdSTS22*	Aradu.ETY3A	A04	Aradu.A04:11112723..11114706	1,983	389	42.86	6.04	32.00	92.72	-0.068	Plastid
25	*AdCHS3*	Aradu.11DJA	A05	Aradu.A05:17517388..17520292	2,904	433	48.14	6.61	42.15	88.08	-0.173	Golgi apparatus
26	*AdCHS4*	Aradu.F4M5D	A05	Aradu.A05:17541681..17543350	1,669	389	42.89	6.76	40.44	90.75	-0.123	Plastid
27	*AdCHS5*	Aradu.WB4GB	A06	Aradu.A06:93250793..93255394	4,601	393	43.23	6.18	38.37	88.58	-0.124	Cytoplasm

* PeanutBase (https://www.peanutbase.org/)

Table S2. Members of CHS and STS families in *Arachis ipaënsis* genome.

Gene						Deduced Protein						Subcellular Localization (Plant-mSubP)
**Nº**	**Nomenclature**	**PeanutBase ID***	**Chromossome**	**Genome position***	**ORF length (bp)**	**Size (aa)**	**MW (kDa)**	**pI**	**II**	**AI**	**GRAVY**	
1	*AiSTS1*	Araip.PF5R6	B01	Araip.B01:9,622,636..9,624,428	1,793	389	42.86	5.80	34.30	92.24	-0.090	Plastid
2	*AiCHS1*	Araip.IA0U9	B03	Araip.B03:11,447,448..11,449,968	2,521	376	41.54	5.70	39.56	88.96	-0.074	Cytoplasm
3	*AiSTS2*	Araip.5S2PS	B04	Araip.B04:2,527,877..2,529,340	1,464	344	37.78	5.64	36.33	95.78	-0.052	Plastid
4	*AiSTS3*	Araip.73H5Y	B04	Araip.B04:2,549,548..2,551,064	1,517	367	40.45	5.32	38.85	95.64	-0.011	Plastid
5	*AiSTS4*	Araip.U7CA5	B04	Araip.B04:11,884,607..11,886,435	1,829	389	42.86	5.80	33.87	92.98	-0.064	Plastid
6	*AiSTS5*	Araip.H6229	B04	Araip.B04:11,935,038..11,948,045	13,008	379	41.48	5.85	33.80	93.11	-0.048	Plastid
7	*AiSTS6*	Araip.25IGU	B04	Araip.B04:11,979,690..11,982,488	2,799	362	39.59	5.78	36.00	96.41	0.009	Plastid
8	*AiSTS7*	Araip.YDG4K	B04	Araip.B04:12,010,115..12,023,432	13,318	387	42.71	5.96	32.36	89.92	-0.125	Cytoplasm
9	*AiSTS8*	Araip.3T4SK	B04	Araip.B04:12,057,904..12,059,388	1,485	387	42.45	5.24	35.00	94.99	-0.028	Plastid
10	*AiSTS9*	Araip.9Z32Z	B04	Araip.B04:12,070,966..12,071,928	963	320	34.87	5.38	34.10	99.03	0.066	Cytoplasm
11	*AiSTS10*	Araip.S9JRR	B04	Araip.B04:12,080,850..12,082,158	1,309	375	41.07	5.43	37.14	93.09	-0.032	Plastid
12	*AiSTS11*	Araip.YF2QF	B04	Araip.B04:12,121,743..12,123,473	1,731	389	42.78	5.96	32.78	91.75	-0.076	Cytoplasm
13	*AiSTS12*	Araip.FM6GG	B04	Araip.B04:12,195,717..12,196,679	963	320	34.98	5.18	36.35	98.72	0.051	Cytoplasm
14	*AiSTS13*	Araip.THJ4F	B04	Araip.B04:12,276,365..12,277,327	963	320	34.93	5.17	37.21	96.28	0.031	Cytoplasm
15	*AiSTS14*	Araip.V9QDS	B04	Araip.B04:12,279,740..12,280,621	882	293	31.80	5.21	35.13	98.81	0.104	Cytoplasm
16	*AiSTS15*	Araip.Z5UEI	B04	Araip.B04:12,285,747..12,286,918	1,172	336	36.78	5.29	36.68	94.29	-0.028	Plastid
17	*AiCHS2*	Araip.B8TJ0	B04	Araip.B04:12,655,555..12,657,109	1,555	389	42.94	6.22	38.62	86.99	-0.136	Plastid
18	*AiSTS16*	Araip.E7BUX	B04	Araip.B04:12,684,969..12,687,006	2,038	389	42.81	7.00	31.78	91.98	-0.075	Plastid
19	*AiSTS17*	Araip.QL51L	B04	Araip.B04:12,730,078..12,731,882	1,805	389	42.84	5.80	34.29	91.98	-0.078	Plastid
20	*AiSTS18*	Araip.TTM09	B04	Araip.B04:12,733,386..12,735,343	1,958	390	42.93	6.14	31.56	92.51	-0.063	Cytoplasm
21	*AiSTS19*	Araip.7P0TV	B04	Araip.B04:12,749,777..12,751,674	1,898	389	42.79	5.71	34.73	93.24	-0.066	Plastid
22	*AiSTS20*	Araip.8ZH5X	B04	Araip.B04:12,802,390..12,804,361	1,972	390	42.90	6.14	32.03	92.26	-0.091	Plastid
23	*AiSTS21*	Araip.62EH4	B04	Araip.B04:12,804,844..12,806,649	1,806	389	42.83	5.80	33.16	91.72	-0.078	Plastid
24	*AiCHS3*	Araip.JD11C	B06	Araip.B06:115,739,106..115,742,862	3,757	393	43.16	6.04	37.88	89.31	-0.106	Cytoplasm
25	*AiSTS22*	Araip.LS9BW	B06	Araip.B06:130,707,884..130,709,906	2,023	390	42.91	6.14	31.56	92.51	-0.056	Plastid
26	*AiSTS23*	Araip.Q8ZS3	B06	Araip.B06:130,711,132..130,713,193	2,062	389	42.79	5.80	32.55	91.72	-0.077	Plastid

* PeanutBase (https://www.peanutbase.org/)

Table S3. Members of CHS and STS families in *Arachis stenosperma* genome.

Gene					Deduced Protein						Subcellular Localization (Plant-mSubP)
Nº	Nomenclature	Chromossome	Genome position*	ORF length (bp)	Size (aa)	MW (kDa)	pI	II	AI	GRAVY	
1	*AsSTS1*	A03	arast.V10309.gnm1.chr01:3,168,004..3,169,589	1,585	389	42.87	5.96	33.42	92.49	-0.090	Plastid
2	*AsCHS1*	A04	arast.V10309.gnm1.chr03:9,122,380..9,124,833	2,453	389	42.95	6.12	40.56	88.77	-0.104	Plastid
3	*AsSTS2*	A04	arast.V10309.gnm1.chr04:1,927,806..1,929,586	1,780	389	42.79	5.67	36.81	93.98	-0.080	Plastid
4	*AsSTS3*	A04	arast.V10309.gnm1.chr04:12519681..12521174	1,496	389	42.80	5.8	33.38	91.98	-0.074	Plastid
5	*AsSTS4*	A04	arast.V10309.gnm1.chr04:13112419..13114200	1,781	391	43.16	6.62	31.10	91.76	-0.090	Plastid
6	*AsSTS5*	A04	arast.V10309.gnm1.chr04:13150290..13152182	1,892	389	42.84	5.80	33.65	91.98	-0.078	Plastid
7	*AsSTS6*	A04	arast.V10309.gnm1.chr04:13157182..13159138	1,956	391	43.08	6.41	32.11	92.76	-0.066	Cytoplasm
8	*AsSTS7*	A04	arast.V10309.gnm1.chr04:13228824..13230645	1,821	391	43.12	6.62	31.10	91.76	-0.084	Cytoplasm
9	*AsSTS8*	A04	arast.V10309.gnm1.chr04:13267540..13269450	1,910	389	42.75	5.95	32.66	90.72	-0.085	Plastid
10	*AsSTS9*	A04	arast.V10309.gnm1.chr04:13308095..13309872	1,777	389	42.85	5.79	34.62	91.75	-0.090	Plastid
11	*AsSTS10*	A04	arast.V10309.gnm1.chr04:13339407..13341439	2,032	389	42.73	5.95	32.83	91.98	-0.081	Plastid
12	*AsSTS11*	A04	arast.V10309.gnm1.chr04:13372266..13374153	1,887	389	42.83	5.74	34.74	91.75	-0.087	Plastid
13	*AsSTS12*	A04	arast.V10309.gnm1.chr04:13384713..13386840	2,127	389	42.82	6.35	32.36	90.98	-0.085	Plastid
14	*AsSTS13*	A04	arast.V10309.gnm1.chr04:13407299..13409432	2,133	388	42.61	5.95	33.99	91.49	-0.065	Plastid
15	*AsSTS14*	A04	arast.V10309.gnm1.chr04:13441600..13443700	2,100	389	42.75	5.66	34.04	92.24	-0.074	Plastid
16	*AsSTS15*	A04	arast.V10309.gnm1.chr04:13480832..13482748	1,916	389	42.76	5.65	33.07	91.49	-0.090	Plastid
17	*AsSTS16*	A04	arast.V10309.gnm1.chr04:13597668..13599765	2,097	389	42.76	5.62	34.00	91.75	-0.086	Plastid
18	*AsSTS17*	A04	arast.V10309.gnm1.chr04:13618402..13620305	1,903	389	42.75	5.95	32.85	91.49	-0.092	Plastid
19	*AsSTS18*	A04	arast.V10309.gnm1.chr04:13784827..13786783	1,956	389	42.82	5.56	32.52	91.75	-0.096	Plastid
20	*AsCHS2*	A04	arast.V10309.gnm1.chr04:13815467..13816885	1,418	389	42.99	6.05	39.03	87.25	-0.161	Cytoplasm
21	*AsSTS19*	A04	arast.V10309.gnm1.chr04:13856902..13858728	1,826	389	42.85	5.74	33.44	92.24	-0.061	Plastid
22	*AsSTS20*	A04	arast.V10309.gnm1.chr04:13927334..13929002	1,668	389	42.85	5.80	31.81	91.72	-0.087	Plastid
23	*AsCHS3*	A04	arast.V10309.gnm1.chr05:19842691..19845569	2,878	389	42.86	6.76	40.44	90.75	-0.121	Cytoplasm
24	*AsCHS4*	A04	arast.V10309.gnm1.chr05:19875719..19877380	1,661	389	42.89	6.76	40.44	90.75	-0.123	Plastid
25	*AsCHS5*	A05	arast.V10309.gnm1.chr06:123369453..123374039	4,586	393	43.22	6.04	37.25	88.58	-0.105	Cytoplasm

* PeanutBase (https://www.peanutbase.org/)

Table S4. Members of CHS and STS families in *Arachis hypogaea* genome.

Gene						Deduced Protein						Subcellular Localization
Nº	Nomenclature	PeanutBase ID*	Chromossome	Genome position*	ORF length (bp)	Size (aa)	MW (kDa)	pI	II	AI	GRAVY	(Plant-mSubP)
1	*AhSTS1*	arahy.TF0YG1	01	Arahy.01:3,140,585..3,143,008	2,424	389	42.89	5.96	34.30	92.49	-0.090	Plastid
2	*AhCHS1*	arahy.UDJX6I	03	Arahy.03:8,632,413..8,635,267	2,855	389	42.92	6.48	40.01	89.77	-0.102	Plastid
3	*AhSTS2*	arahy.GQLW6A	04	Arahy.04:1,920,212..1,922,274	2,063	389	42.79	5.55	36.81	93.98	-0.08	Plastid
4	*AhSTS3*	arahy.M1C18U	04	Arahy.04:10,843,366..10,845,412	2,047	389	42.77	5.66	32.96	91.72	-0.062	Plastid
5	*AhSTS4*	arahy.63FKND	4	Arahy.04:10,850,176..10,851,915	1,740	364	39.76	5.85	33.42	97.25	0.011	Plastid
6	*AhSTS5*	arahy.1B4KK2	04	Arahy.04:10,899,699..10,901,838	2,140	389	42.81	5.66	35.39	92.24	-0.071	Plastid
7	*AhSTS6*	arahy.JV52YF	04	Arahy.04:10,905,965..10,908,058	2,094	390	43.00	6.35	30.66	93.00	-0.061	Cytoplasm
8	*AhSTS7*	arahy.NS7LJX	04	Arahy.04:10,949,941..10,952,057	2,117	389	42.92	5.79	32.85	93.24	-0.073	Plastid
9	*AhSTS8*	arahy.BLK73U	04	Arahy.04:10,956,032..10,958,093	2,062	389	42.83	5.79	32.65	91.75	-0.091	Plastid
10	*AhSTS9*	arahy.A621IZ	04	Arahy.04:10,987,549..10,989,534	1,986	389	42.86	5.79	34.62	91.75	-0.093	Plastid
11	*AhSTS10*	arahy.3DZG8W	04	Arahy.04:10,999,412..11,001,499	2,088	389	32.87	6.35	31.62	90.23	-0.083	Plastid
12	*AhSTS11*	arahy.BCQ67I	04	Arahy.04:11,108,405..11,110,614	2,210	389	42.80	6.62	29.66	92.24	-0.086	Plastid
13	*AhSTS12*	arahy.ME0HAD	04	Arahy.04:11,170,022..11,171,957	1,936	367	40.03	5.50	37.32	95.12	-0.031	Plastid
14	*AhSTS13*	arahy.KVQE6R	04	Arahy.04:11,195,163..11,197,400	2,238	389	42.76	5.65	33.07	91.49	-0.090	Plastid
15	*AhSTS14*	arahy.EPR46C	04	Arahy.04:11,288,907..11,291,272	2,366	389	42.85	5.56	33.13	91.75	-0.098	Plastid
16	*AhSTS15*	arahy.BDJ8VI	04	Arahy.04:11,393,637..11,404,953	11,317	387	42.64	6.35	32.43	91.71	-0.081	Cytoplasm
17	*AhSTS16*	arahy.1N9K04	04	Arahy.04:11,437,162..11,438,875	1,714	390	43.03	6.55	31.74	93.00	-0.069	Plastid
18	*AhSTS17*	arahy.ZK4XM6	04	Arahy.04:11,458,470..11,460,820	2,351	390	42.95	5.95	29.39	93.00	-0.057	Plastid
19	*AhSTS18*	arahy.U6WJF3	04	Arahy.04:11,461,924..11,465,577	3,654	389	42.82	5.70	33.64	91.47	-0.074	Plastid
20	*AhCHS2*	arahy.K0UY8K	05	Arahy.05:38,961,309..38,963,310	2,002	389	42.89	6.76	40.44	90.75	-0.123	Plastid
21	*AhSTS19*	arahy.95I963	11	Arahy.11:10,150,770..10,153,195	2,426	389	42.86	5.80	34.30	92.24	-0.090	Plastid
22	*AhCHS3*	arahy.0FI6RG	13	Arahy.13:11,844,796..11,847,164	2,369	380	42.00	5.71	37.91	87.79	-0.113	Plastid
23	*AhSTS20*	arahy.303ZYM	14	Arahy.14:2,637,052..2,638,957	1,906	389	42.88	5.89	34.99	92.24	-0.096	Plastid
24	*AhSTS21*	arahy.UKNP9M	14	Arahy.14:2,660,308..2,662,362	2,055	389	42.82	5.67	36.43	92.98	-0.083	Plastid
25	*AhSTS22*	arahy.GTM8IY	14	Arahy.14:12,410,452..12,412,394	1,943	389	42.80	5.80	33.38	91.98	-0.072	Plastid
26	*AhSTS23*	arahy.H6K3LP	14	Arahy.14:12,421,243..12,423,495	2,253	390	42.96	6.04	34.01	92.00	-0.063	Cytoplasm
27	*AhSTS24*	arahy.HGK3FC	14	Arahy.14:12,461,877..12,464,675	2,799	362	39.59	5.78	36.00	96.41	0.009	Plastid
28	*AhSTS25*	arahy.I9DV29	14	Arahy.14:12,495,040..12,496,987	1,948	389	42.86	5.80	33.16	91.47	-0.085	Plastid
29	*AhSTS26*	arahy.HC1E0U	14	Arahy.14:12,555,149..12,557,112	1,964	389	42.85	5.96	31.71	92.24	-0.081	Plastid
30	*AhSTS27*	arahy.V739AW	14	Arahy.14:12,567,832..12,569,930	2,099	389	42.74	6.61	31.33	93.50	-0.061	Plastid
31	*AhSTS28*	arahy.9Q60JN	14	Arahy.14:12,574,839..12,576,929	2,091	389	42.83	5.80	35.67	91.75	-0.070	Plastid
32	*AhSTS29*	arahy.I6S6H6	14	Arahy.14:12,619,232..12,621,155	1,924	389	42.78	5.96	32.78	91.75	-0.076	Cytoplasm
33	*AhSTS30*	arahy.3Z4TCL	14	Arahy.14:12,660,036..12,668,048	8,013	404	44.45	5.81	38.88	89.06	-0.118	Cytoplasm
34	*AhSTS31*	arahy.A0U9FM	14	Arahy.14:12,774,557..12,776,867	2,311	389	42.80	5.66	34.38	91.72	-0.088	Plastid
35	*AhSTS32*	arahy.WR54RW	14	Arahy.14:12,781,801..12,783,773	1,973	389	42.83	5.65	33.00	90.49	-0.103	Plastid
36	*AhSTS33*	arahy.HQXJ4B	14	Arahy.14:12,815,811..12,817,783	1,973	389	42.83	5.65	33.00	90.49	-0.103	Plastid
37	*AhSTS34*	arahy.DV6K6C	14	Arahy.14:12,867,967..12,869,939	1,973	389	42.83	5.65	33.00	90.49	-0.103	Plastid
38	*AhSTS35*	arahy.QVKQ5Y	14	Arahy.14:12,922,134..12,924,354	2,221	389	42.78	5.65	33.07	91.49	-0.090	Plastid
39	*AhSTS36*	arahy.2T7FQZ	14	Arahy.14:12,965,692..12,967,918	2,227	389	42.76	5.76	33.07	91.49	-0.090	Plastid
40	*AhSTS37*	arahy.4IBX4Q	14	Arahy.14:13,017,212..13,019,244	2,033	389	42.76	5.65	34.09	92.24	-0.090	Plastid
41	*AhCHS4*	arahy.GSLK7G	14	Arahy.14:13,196,334..13,197,950	1,617	389	42.94	6.22	38.62	86.99	-0.136	Plastid
42	*AhSTS38*	arahy.YZ4W1Q	14	Arahy.14:13,226,398..13,228,582	2,185	389	42.81	7.00	31.78	91.98	-0.075	Plastid
43	*AhSTS39*	arahy.F4M9UE	14	Arahy.14:13,273,378..13,275,397	2,020	389	42.84	5.80	34.29	91.98	-0.078	Plastid
44	*AhSTS40*	arahy.Z2RDVM	14	Arahy.14:13,276,783..13,279,100	2,318	390	42.93	6.14	31.56	92.51	-0.063	Cytoplasm
45	*AhSTS41*	arahy.R1Z4C8	14	Arahy.14:13,292,704..13,294,726	2,023	389	42.79	5.71	34.73	93.24	-0.066	Plastid
46	*AhSTS42*	arahy.5YH4B6	14	Arahy.14:13,324,600..13,326,847	2,248	390	42.99	7.00	30.49	92.00	-0.076	Cytoplasm
47	*AhSTS43*	arahy.VU1QU6	14	Arahy.14:13,347,467..13,349,785	2,319	390	42.90	6.14	32.03	92.26	-0.091	Plastid
48	*AhSTS44*	arahy.6JHG9N	14	Arahy.14:13,350,994..13,353,014	2,021	389	42.83	5.80	33.16	91.72	-0.078	Plastid
49	*AhCHS5*	arahy.6J3HHE	15	Arahy.15:18,572,663..18,574,554	1,892	389	42.99	7.10	39.94	91.00	-0.131	Plastid
50	*AhCHS6*	arahy.QZN0LQ	15	Arahy.15:18,595,150..18,597,004	1,855	389	42.99	7.10	39.94	91.00	-0.131	Plastid
51	*AhSTS45*	arahy.PUDN4D	16	Arahy.16:147,469,931..147,472,126	2,196	390	42.92	6.14	31.73	92.77	-0.055	Plastid
52	*AhSTS46*	arahy.3WH5A3	16	Arahy.16:147,473,252..147,475,265	2,014	389	42.80	5.80	32.55	91.72	-0.077	Plastid

* PeanutBase (https://www.peanutbase.org/)
